# Approaching intrinsic dynamics of MXenes hybrid hydrogel for 3D printed multimodal intelligent devices with ultrahigh superelasticity and temperature sensitivity

**DOI:** 10.1038/s41467-022-31051-7

**Published:** 2022-06-14

**Authors:** Haodong Liu, Chengfeng Du, Liling Liao, Hongjian Zhang, Haiqing Zhou, Weichang Zhou, Tianning Ren, Zhicheng Sun, Yufei Lu, Zhentao Nie, Feng Xu, Jixin Zhu, Wei Huang

**Affiliations:** 1 Frontiers Science Center for Flexible Electronics (FSCFE), Xi’an Institute of Flexible Electronics (IFE), Xi’an Institute of Biomedical Materials & Engineering, Northwestern Polytechnical University (NPU), Xi’an, PR China; 2 State Key Laboratory of Solidification Processing, Center of Advanced Lubrication and Seal Materials, Northwestern Polytechnical University (NPU), Xi’an, PR China; 3 Key Laboratory of Low-Dimensional Quantum Structures and Quantum Control of Ministry of Education, Key Laboratory for Matter Microstructure and Function of Hunan Province, Department of Physics and Synergetic Innovation Center for Quantum Effects and Applications, Hunan Normal University, Changsha, PR China; 4 School of Chemistry and Chemical Engineering, Northwestern Polytechnical University (NPU), Xi’an, PR China; 5 State Key Laboratory of Fire Science, University of Science and Technology of China, Hefei, PR China; 6 Institute of Advanced Materials (IAM), Key Laboratory of Institute of Advanced Materials, Nanjing Tech University (NanjingTech), Nanjing, PR China

**Keywords:** Polymers, Polymers, Sensors and biosensors

## Abstract

Hydrogels are investigated broadly in flexible sensors which have been applied into wearable electronics. However, further application of hydrogels is restricted by the ambiguity of the sensing mechanisms, and the multi-functionalization of flexible sensing systems based on hydrogels in terms of cost, difficulty in integration, and device fabrication remains a challenge, obstructing the specific application scenarios. Herein, cost-effective, structure-specialized and scenario-applicable 3D printing of direct ink writing (DIW) technology fabricated two-dimensional (2D) transition metal carbides (MXenes) bonded hydrogel sensor with excellent strain and temperature sensing performance is developed. Gauge factor (GF) of 5.7 (0 − 191% strain) and high temperature sensitivity (−5.27% °C^−1^) within wide working range (0 − 80 °C) can be achieved. In particular, the corresponding mechanisms are clarified based on finite element analysis and the first use of in situ temperature-dependent Raman technology for hydrogels, and the printed sensor can realize precise temperature indication of shape memory solar array hinge.

## Abstract (web summary)

Cost effective device fabrication of powerful hydrogel sensors remains challenging. Here, the authors propose a cost-effective and structure-specialized direct ink writing technique for the fabrication of two-dimensional MXene bonded hydrogel sensors with excellent strain and temperature sensing performance.

## Introduction

The popularization of scientific and technology globalization promotes innovation-driven international cooperation, speeding up efforts for achieving scientific and technological prosperity of flexible electronics^1–4^. As an indispensable medium for detecting external stimulus in flexible electronics integration system, flexible sensors with high sensitivity and rapid response convert physical or chemical signals into electrical outputs, which have been utilizing in electronic skins and human–machine interactions^5,6^. In particular, polymer hydrogels are widely developed owing to the facile processing of materials and simple fabrication of devices, which have been proven feasible for resistive strain and temperature sensors^7–11^.

For filled-type hydrogels applied into flexible sensors, polymer matrices provide stable three-dimensional (3D) supports, and conductive fillers which can be mainly divided into electrolytes and nanomaterials with various dimensions give the system satisfactory conductivity^12,13^. The addition of electrolytes presents extraordinary conductivity, water retention and frost resistance, but the resultant ionic conductive networks are hardly switched under external stimulus, resulting in relatively lower sensitivity^14,15^. In contrast, conductive nanofillers respond to strain or temperature as a result of the general disconnection principle and the effect of heat on carrier mobility^16,17^. Generally, metal-based nanoparticles reduce the payload of flexible integrated system because of the higher density, and carbon-based materials with large specific surface area and lower density need to be added in large amounts for achieving suitable conductivity and sensitivity, which is detriment to the toughness and strain range, failing to meet the portability requirements of flexible electronics^18–22^. In comparison, the emerging 2D transition metal carbides (MXenes) are possessed with metallic conductivity and higher specific surface area, together with the amelioration of stability in oxygen, which have been developed as revolutionary materials used in electrochemistry^10,11,23–27^. However, the ambiguity in the response mechanism of MXenes based hydrogel sensors inhibits the deep understanding and further employment of multicomponent polymer hydrogel composites^28–30^. Moreover, the fabrication method, processing cost of sensors and multi-functionalization of flexible sensing integration systems remain challenging fields, which further impede the development of specific application scenarios as well^31,32^.

In view of that, this work employed a facile and cost-effective materials extrusion-based 3D printing technology of direct ink writing (DIW) and solvent replacement to fabricate structure-specialized, and scenario-applicable MXenes bonded polyurethane/polyvinyl alcohol (PU/PVA) hydrogel with excellent strain and temperature sensing performance. The optimal loadings of MXenes and glycerol were determined through investigating the influence of each component on the mechanical strength and MXenes on the strain sensing performance. Further, different strain sensitivities were obtained by printing hydrogels with various structure-specialized patterns, one of those samples was selected for detailed strain and temperature sensing analysis. The printed hydrogel sensor possesses excellent sensing performance, the mechanical behaviors and strain sensing mechanism during stretching were deeply studied by the finite element analysis methods. In particular, the in situ temperature-dependent Raman technology was firstly employed to reveal the temperature response mechanism of thermal-induced tunneling effect in the hydrogel sensor. In addition, owing to the high sensitivity and wide working range, the sensor was not only used for human motion recognition based on the strain response, but also utilized for realizing precise temperature indication of shape memory solar array hinge at aerospace domain.

## Results

### Fabrication and structure of hydrogels and raw materials

**Fig. 1:**
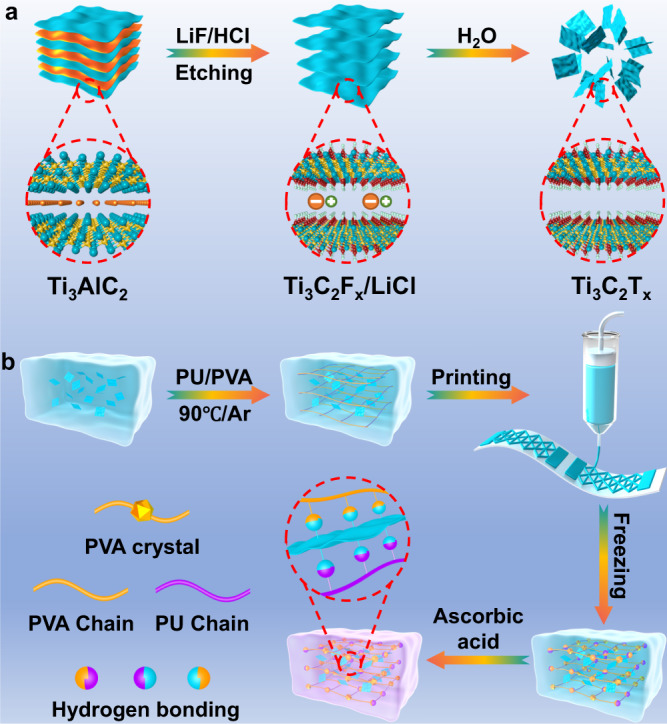
Synthesis of MXenes and hydrogel. **a** Schematic illustration for the preparation of Ti_3_C_2_T_x_ flakes. **b** Direct ink writing (DIW) printing of transition metal carbides (MXenes) bonded polyurethane/polyvinyl alcohol (PU/PVA) hydrogel.

Figure 1a depicts the schematic demonstration of MXenes preparation. Generally, fluoride-based salt etching system was applied here for purpose of reducing risks of concentrated hydrogen fluoride (HF) dilution^33^. Typically, LiF crystals were mildly dissolved in hydrochloric acid (HCl) aqueous solution, resulting in gradual in situ formation of HF with lower concentration. Under protection of low temperature, the mixture of LiF and HCl was gradually blended with Ti_3_AlC_2_ powders, retarding excessive air bubs generated from exothermic reaction between the powders and etchants. The obtained black suspension was further treated at 35 °C for ensuring sufficient etching and avoiding extra oxidation of fresh MXenes. The etched products were washed and intercalated for obtaining multilayered MXenes, which were peeled off with the assistance of sonication and argon (Ar) protection. Finally, long-time centrifugation isolated the delaminated Ti_3_C_2_T_x_ flakes from multilayered aggregates.

In the presence of electronegative groups on Ti_3_C_2_T_x_, the corresponding polymer hydrogel networks were constructed according to the illustration shown in Fig. 1b. Typically, lyophilized Ti_3_C_2_T_x_ flakes were dispersed homogenously before adding glycerol and polymers. The black blend was heated to dissolve PVA under Ar atmosphere, avoiding unnecessary oxidation of Ti_3_C_2_T_x_ caused by high temperature. The obtained liquid precursor was injected into syringe and printed through DIW printer. In the process of printing, as recorded in Supplementary Movie 1, the viscous precursor in the syringe which had been pretreated for de-bubbling was continuously extruded through the needle driven by air pump pressure, and deposited on polyethylene terephthalate (PET) substrate with a certain height from the needle. In addition, extrusion pressure, printing height and moving speed had been constantly optimized, achieving the continuity of the whole extruding process and the integrity of the printed patterns designed by computer. Owing to the favorable processing parameters, the structure of the extruded precursor is well-maintained. The following gelation was carried out by freeze-thaw treatment, and the ionized L-ascorbic acid anions can bonded with titanium atoms on the edge of Ti_3_C_2_T_x_^34^. As a result, the printed hydrogel could be directly adopted for sensing application without extra encapsulation.

**Fig. 2:**
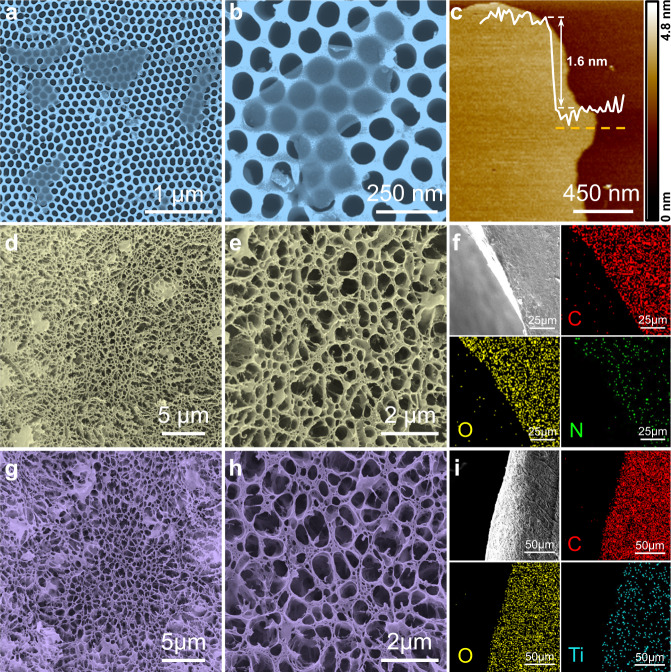
Morphology and structure of materials. **a**, **b** Scanning electron microscope (SEM) morphology and (**c**) atomic force microscope (AFM) image of Ti_3_C_2_T_x_ flakes. Microstructure of (**d**, **e**) pristine lyophilized gel with (**f**) corresponding elements distribution. Microstructure of (**g**, **h**) Ti_3_C_2_T_x_ loaded lyophilized gel with (**i**) elements distribution.

In addition, the morphology and structure of the as-mentioned materials were characterized through high-resolution field-emission scanning electron microscope (FESEM). As demonstrated in Supplementary Fig. 1a, unetched Ti_3_AlC_2_ powders present distinctive layered stacked aggregates, which are consistent with the inner microscopic structure of Al atoms sandwiched by Ti_3_C_2_ sheets. After etching and delamination, from Supplementary Fig. 1b, the colloidal solution of delaminated Ti_3_C_2_T_x_ flakes present obvious Tyndall effect. The Ti_3_C_2_T_x_ flakes exhibit smooth 2D nanosheets morphology with the lateral size from 0.4 to 1 μm shown in Fig. 2a, b. From atomic force microscope (AFM) image (Fig. 2c), the obtained Ti_3_C_2_T_x_ flakes present smooth surface with the average thickness of 1.6 nm, which is in accordance with the thickness of Ti_3_C_2_T_x_ bilayer structure^33,35–38^. To investigate the conductivity of Ti_3_C_2_T_x_, the flakes were assembled to a homogeneous membrane with 7 μm thickness via vacuum filtration, and the resultant conductivity was measured at 7.257 × 10^4^ S m^−1^, presenting extraordinary electrical conductivity. In addition, the crystal structure of Ti_3_AlC_2_ powders and Ti_3_C_2_T_x_ were identified through X-ray diffraction (XRD), an obviously shift of (002) peak at 9.45° in Ti_3_AlC_2_ switching to 6.3° of Ti_3_C_2_T_x_ can be noted (Supplementary Fig. 2), which might be resulting from the removal of Al from Ti_3_AlC_2_, the implement of intercalation, and the introduction of surface terminal groups including −O and –OH. Moreover, the chemical components of Ti_3_C_2_T_x_ were explored through X-ray photoelectron spectroscopy (XPS), C–1*s* were fitted in Supplementary Fig. 3a, the peak at 282.45 eV in the C–1*s* spectrum indicates the Ti–C frameworks originated from Ti_3_AlC_2_ are preserved completely. In the course of etching, graphitic C–C bonds were also generated according to the fitted peak at 284.6 eV. In the meantime, located at 286.75, 285.35, and 283.3 eV, the existence of C=O, C–O and C–Ti–O bonds reveals that the termination reaction had been implemented during etching and delamination, which can be further proved from the Ti–2*p* peaks in Supplementary Fig. 3b. Specifically, the peaks at 455.5 eV of Ti–2*p*_3/2_ and 461.65 eV of Ti–2*p*_1/2_ are assigned to Ti–C bonds. Located at 456.25 and 462.65 eV, the peaks associated with other peaks at 457.15 eV (Ti–2*p*_3/2_) and 463.7 eV (Ti–2*p*_1/2_) prove the bonding of C–Ti–O and C–Ti–OH, respectively. Note that a weak peak at 488.8 eV of TiO_2_ can be detected, illustrating the slight oxidation of Ti_3_C_2_T_x_ flakes occurred during the delamination process.

In order to get the detailed internal microstructure of the hydrogel networks, the printed hydrogel was lyophilized after solvent replacement. As shown in Fig. 2d, e, the printed pristine hydrogel exhibits random oriented porous network structure with hierarchical pore size distribution. The cross-sectional morphology of the printed hydrogel with the corresponding energy dispersive X-ray spectroscopy (EDS) elemental mapping were shown in Fig. 2f, which confirm the homogeneous distribution of C, O and N elements. Illustrated from the EDS element distribution, the relative content of N element is less than that of C and O, which means that the contents of hard domains composed of nitrogen-containing groups in PU network is less than the soft domains, influencing the mechanical strength of the hydrogel. Similarly, after added 0.25 wt% Ti_3_C_2_T_x_ flakes into the precursor, the printed hydrogel displays unoriented porous framework with random pore size distribution as that of the pristine hydrogel (Fig. 2g, h), and EDS of C, O and Ti element (Fig. 2i) illustrates that Ti_3_C_2_T_x_ flakes are distributed homogenously. Furthermore, the construction of hydrogel frameworks was revealed through spectroscopic analysis. Supplementary Fig. 4 demonstrates the XRD patterns of glycerol loaded hydrogel with different components, pristine PVA gel presents a strong peak at 2*θ* of 19.5° owing to partial crystallization of PVA segments under sub-zero temperature, which can be preserved after loading PU and Ti_3_C_2_T_x_ flakes. In addition, Fourier transform infrared spectroscopy (FTIR) from Supplementary Fig. 5 proves the chemical bonding of the hydrogel. The absorption peak of –OH groups at 3365 cm^−1^ and C–O bond at 1099 cm^−1^ of PVA/H_2_O gel obviously shift to 3305 and 1037 cm^−1^ after successively adding PU, glycerol and Ti_3_C_2_T_x_ flakes, meaning that formation of supramolecular hydrogen bonding networks between PVA, PU, and Ti_3_C_2_T_x_. As a consequence, the printed hydrogel was fabricated by PVA crystal domains and hydrogen bonds among each component. Furthermore, the glycerol works as non-electrolyte humectant that enables the water retention capacity of the hydrogel, which can be supported by Supplementary Fig. 6. Obviously, the solvent content of glycerol loaded hydrogel can be maintained at 94% under 54% relative humidity (RH) after 20 days, while the gel without glycerol presents rapid dehydration within 4 days, hence the addition of glycerol is beneficial to maintain the stability of the hydrogel.

### Formula optimization and performance comparison

Since the strain sensing range of the hydrogel is highly depends on its mechanical properties, the tensile strengths, elongations at break (strain), compression-recovery behaviors and elastic modulus of each sample were evaluated with a stretching or compression speed of 1 mm/s. As presented in Supplementary Fig. 7a, compared with pure PVA system, the incorporation of PU dramatically enhanced the tensile strength from 0.025 to 0.16 MPa with the elongations at break being raised from 105% to 148%. Similarly, the compressive strength at 60% strain was increased from 0.14 to 0.16 MPa (Supplementary Fig. 7b, c). Meanwhile, the corresponding elastic modulus were heightened from 0.029 to 0.114 MPa (Supplementary Fig. 7d). On the basis of the synergy between PU and PVA, the mechanical properties can be further strengthened by introduced glycerol and Ti_3_C_2_T_x_ MXenes. As illustrated in Supplementary Fig. 8a, the tensile strengths were improved evidently from 0.16 to 1.4 MPa with the corresponding strain ranging from 148% to 424%, meanwhile, the compressive strength was improved to 0.7 MPa (Supplementary Fig. 8b–e) and the elastic module was increased to 0.33 MPa when the glycerol loading reaching 60 wt% in PU/PVA hydrogel (Supplementary Fig. 8f). Further, the addition of Ti_3_C_2_T_x_ flakes can reinforce the hydrogel as well. From Supplementary Fig. 9a, based on the pristine PU/PVA hydrogel involving glycerol, it can be found that the tensile strengths were stepped up from 1.4 to 2.28 MPa when adding 0.25 wt% Ti_3_C_2_T_x_, showing relatively superior mechanical strength (Supplementary Fig. 10b). Noticeably, an apparent decline of stresses and strains was observed from the hydrogel with further increased the Ti_3_C_2_T_x_ contents, resulting in peak values at 0.25 wt% Ti_3_C_2_T_x_ loading. However, with additional Ti_3_C_2_T_x_ loadings still comes to great compression strength which was raised from 0.88 MPa at 0.1 wt% Ti_3_C_2_T_x_ to 1.8 MPa at 2 wt% Ti_3_C_2_T_x_ under 60% compressive strain (Supplementary Fig. 9b–f), and the maximum elastic module of 0.84 MPa was achieved through adding 2 wt% Ti_3_C_2_T_x_ (Supplementary Fig. 10a). For 0.25 wt% Ti_3_C_2_T_x_ loaded hydrogel with peak tensile strength, dynamic mechanical analysis (DMA) was performed in comparison to 0 wt% Ti_3_C_2_T_x_ loading. As presented in Supplementary Fig. 11a, the storage modulus (*E*′) was significantly improved from 2827 MPa to 6514 MPa at −150 °C when adding 0.25 wt% Ti_3_C_2_T_x_, proving the superior anti-deformation ability of Ti_3_C_2_T_x_ hydrogel. Then sharp decline happened to *E*′ of both samples from −100 to −55 °C, meaning that transition of two gel samples from glassy to elastic state occurred. The loss modulus (*E″*) was also increased from 51.81 MPa to 125.2 MPa at −150 °C as a result of loading Ti_3_C_2_T_x_ (Supplementary Fig. 11b), and a dramatic growth of peak *E″* can be observed (from 452.5 to 937.6 MPa) within the transition interval. Although the addition of Ti_3_C_2_T_x_ intensified the energy dissipation, the gap between *E*′ and *E″* is even more pronounced. Both gel samples transited to elastic state during temperature rising, hence the glass transition temperature (T_g_) was measured as −72 °C of 0.25 wt% Ti_3_C_2_T_x_ hydrogel and −65 °C of Ti_3_C_2_T_x_ unloaded sample (Supplementary Fig. 11c). therefore, the enhancement of *E*’ and *E*” as well as the decrease of T_g_ revealing that superb elasticity of the hydrogel can be achieved through introducing Ti_3_C_2_T_x_.

The anti-deformation ability of the hydrogel mainly depends on the existence of hydrogen bonds, van der Waals force, and other chemical bonding^39,40^. PU and PVA chains are arranged regularly with fewer branched chains and smaller chain spacing, which promotes the formation of chain entanglements. Meanwhile, both glycerol and Ti_3_C_2_T_x_ can form hydrogen bonds with polymer chains, which effectively increases the physical cross-linking density of the hydrogel, resulting in the enhancement of tensile and compressive strength as well as elastic modulus. The addition of glycerol improves the tensile strain owing to sufficient chains slippage, but the introduction of Ti_3_C_2_T_x_ restricts the free volume and flexibility of the polymer chains. As a consequence, the hydrogel hardly can be prone to fracture, leading to a slight decrease in strain. Moreover, the stress can be dispersed to other molecular chains through the cross-linking points (Ti_3_C_2_T_x_) when single chain is subjected external force. If the single cross-linked chain breaks, the strength of the hydrogel can still be supported by other chains. Note that excessive Ti_3_C_2_T_x_ loadings always lead to stress concentration, weakening the tensile stress of the hydrogel.

The investigation of mechanical properties presents that the optimal stretching performance can be achieved through adding 0.25 wt% Ti_3_C_2_T_x_, which encourages the further exploration about the effect of Ti_3_C_2_T_x_ loadings on strain sensing. As demonstrated in Supplementary Fig. 12, the voltage–current curves reveals that the resistance of the devices continued to decline from 0.71 MΩ of unloaded sample to 0.42 MΩ of 2 wt% Ti_3_C_2_T_x_ loaded hydrogel, meaning the conductivity of the specimens can be controlled by the Ti_3_C_2_T_x_ loadings. The relationship between the relative resistance change (Δ*R*/*R*_0_) and the corresponding strain change (Δ*ε*) of the hydrogel samples with different MXenes contents was plotted in Supplementary Fig. 13, where Δ*R*, *R*_0_ Δ*ε* means the resistance change, initial resistance, and the strain change. The gauge factor (GF) that represents the sensitivity, was calculated by fitting the slope of the strain–resistance change curves. Specifically, the GF of pristine hydrogel was determined as 2.09 in a strain range of 0–400%. Whereas with an increased MXenes contents, the maximum of GF reached 5.03 in the strain range of 155–375% with 0.25 wt% MXenes contents. Even within the strain range of 0–155%, the sample can still deliver a GF of 2.96. Further increasing the MXenes contents will lead to the decrease of GF, for which a GF of 2.1 was observed on the hydrogel with 2 wt% MXenes contents. As the only conductive component in the hydrogel, the continuity of MXenes network directly affects the total resistance, i.e., insufficient MXenes content breaks the conductivity, while excessive amounts of MXenes results in an oversaturated conductivity, note that glycerol only provides sufficient hydroxyl groups for hydrogen bonding as a result of non-electrolyte performance. Therefore, with an overall consideration of mechanical strength and GF, the hydrogel with 0.25 wt% Ti_3_C_2_T_x_ MXenes loading was chosen for printing in different patterns with same gauge distance (30 mm) to further evaluate the strain sensitivity performance. Interestingly, the diversity of printed structure brings about differences in GF, which can meet the requirement of various application scenarios. Here, the multi triangle pattern from Supplementary Fig. 14g with highest GF (5.7) was selected for subsequent strain and temperature response analysis.

### Strain and temperature sensing behaviors

**Fig. 3:**
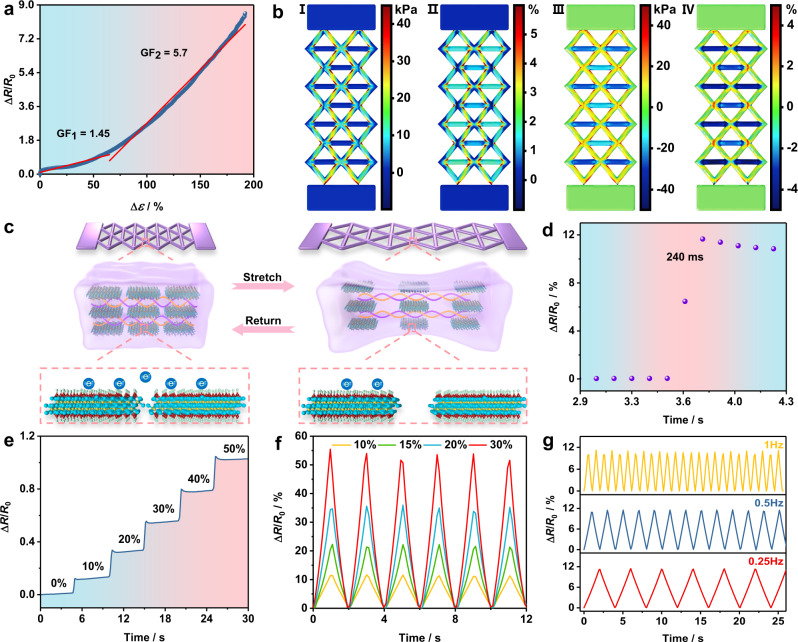
Strain sensing performance of printed hydrogel. **a** Gauge factor (GF) and (**b**) stress–strain distribution simulation of stretched sensor. I Stress and II strain distribution along stretching direction, III stress and IV strain distribution in horizontal direction. **c** Schematic illustration of sensing mechanism. **d** Response time of the printed sensor. Relative resistance change (Δ*R*/*R*_0_) under (**e**) step strains, (**f**) six cyclic stretching-releasing with frequency of 0.5 Hz, and (**g**) different frequencies with 10% strain.

For strain sensing performance, Fig. 3a shows the corresponding sensitivity within different strain ranges. The results present a nonlinear response between Δ*R*/*R*_0_ and Δ*ε*, where GF within the strain of 0–66% is fitted as 1.45, then rises to 5.7 within 66–191% strain range. For investigating the sensing mechanism, the stress–strain distribution model under small strain was established at first, and the simulation results are illustrated in Fig. 3b. Compared to unstretched model in Supplementary Fig. 15, it is obvious that stress and strain are mainly concentrated on the hypotenuses and intersections of the pattern along stretching direction (Fig. 3b, I, II), as a result of that the cross-sectional area of these hypotenuses are compressed and the length is elongated under gradually increased strain. Meanwhile, the stress and strain distribution in horizontal direction are concentrated on the base edges of the pattern, it shows that the edges are slightly bended during the stretching, and provides structural stability to the pattern, which in turn leads to noticeable stress and strain concentration along the stretching direction.

Based on the simulation results, the response mechanism can be mainly summarized as the structure transformation of the conductive network constructed by Ti_3_C_2_T_x_ flakes, which is illustrated in Fig. 3c. Within a narrow strain range at early stage, relative displacement occurs among the flakes, but free electrons are able to pass through the breakpoints of the conductive network according to tunnel effect, leading to a small resistance change and lower GF. The entire conductive network can be disintegrated further as a result of the fillers spacing continuing to expand, even if a few flakes could be compressed together again in the vertical direction to the surface of the sensor, the expansion along the stretching direction still dominates, resulting in further blocking of electrons transmission^41^. A sharp rise of the resistance and higher GF can be obtained, demonstrating a nonlinear response of the whole electromechanical process.

Figure 3d defines the response time at 10% strain of the sensor. In order to eliminate the errors of duration time required to reach the strain, the stretching speed of the machine was turned up to a maximum of 17 mm/s, hence the actual response time was measured as 240 ms, indicating the fast response of the sensor. Figure 3e presents the resistance response under a step strain from 0 to 50% with the duration of 5 s. It can be clearly observed that the growth in Δ*R*/*R*_0_ differs from the linear increase trend of strain, which is in agreement with the nonlinear response in Fig. 3a. In addition, Δ*R*/*R*_0_ quickly increased then remained at a stable level during the stretching, meaning the steady signal output and rapid detecting different strains of the sensor. At a frequency of 0.5 Hz, the sensor undergone 6 stretch-release cycles at different strains (10%, 15%, 20% and 30%, respectively), and the corresponding resistive responses are listed in Fig. 3f, similarly, the responses at different frequencies (0.25, 0.5 and 1 Hz at 10% strain, respectively) were exhibited in Fig. 3g, indicating the sensor can be used for various strain induced application scenarios.

For investigating the cyclic stability of the device, the sensor was subjected to over 5000 stretching-releasing cycles (10% strain, 0.5 Hz), the corresponding signal output was demonstrated in Supplementary Fig. 16, presenting a reliable cyclic stability and duration with a slight increase of resistance. The gradual change of the resistance can be attributed to slight water loss and polymer chains conformation conversion^42^. Generally, the entire conductive network returns to original state when the applied strains are released. However, a few hydrogen bonds can be irreversibly destroyed via polymer chains slippage during the deformation. In addition, from the compression-recovery results discussed before, the polymer hydrogel with various components all present obvious hysteresis circles, which can be intensified under increased compressive strains. The hysteresis of rigid polymer chains is significantly weaker than that of flexible chains. The occurrence of hysteresis is often accompanied by the appearance of mechanical loss. The external force needs to change the chain configurations and overcome the intrinsic friction during the movement of chains when polymer stretching or compressing. Retracting the force, the deformed chains re-coil with frictional resistance being offset during chains motion. Generally, with great frictional resistance comes to serious hysteresis and mechanical loss, the latter also depends on the chains structure. Polymer chains with fewer side groups present less frictional resistance and mechanical loss, but the side group with stronger polarity will lead to the opposite result. Specifically, driven by alternating stress at a specific frequency, chains conformation of PU and PVA can be transformed through internal friction, meaning the chains motion fails to timely match the change of stress. Even the effect of dynamic mechanical relaxation including internal friction and mechanical loss can be slightly weakened through the presence of less hard domains on PU chains, the hysteresis resulted from PVA chains still occurs during deformation-recovery cycles. As a consequence, irreversible relative displacements between Ti_3_C_2_T_x_ flakes and polymer chains can be generated after removing the stress, leading to the slight variation of initial and final resistance. The printed sensor with stable duration can be employed to detect human motion. Specifically, illustrated in Supplementary Fig. 17, the sensor was attached on the wrist by commercial medical tape for monitoring joint bending, the straightened wrist was bent upwards with the sensor being compressed, resulting in a decline of resistance and negative responses of Δ*R*/*R*_0_. Next, the wrist bent to the opposite direction immediately and returned to the initial state again, and the sensor was recovered and stretched. As a result, Δ*R*/*R*_0_ presents a falling and rising signal then returns to a stable state, indicating the potential application on human health monitoring.

**Fig. 4:**
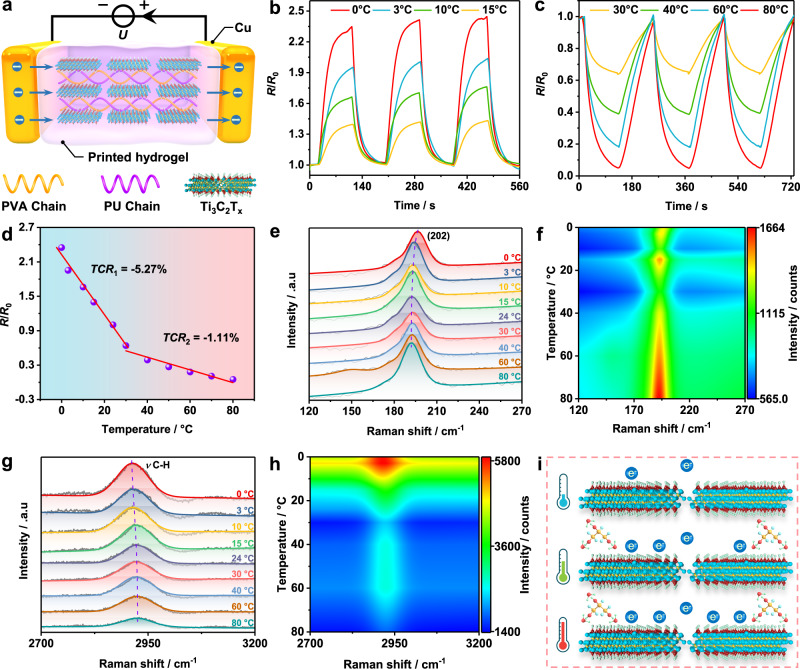
Temperature responses of the printed sensor. **a** Schematic illustration of temperature sensing. **b**, **c** Resistance changes under different temperature (starting temperature: 24 °C), and (**d**) the resultant Temperature coefficient of resistance (*TCR*) for the sensor. Temperature-dependent Raman spectra of (**e**, **f**) Ti_3_C_2_T_x_ and (**g**, **h**) printed thermistor. **i** Temperature sensing mechanism of the printed thermistor.

Similar to the strain sensors fabrication, from Fig. 4a, copper electrodes were attached to the hydrogel for ensuring the effective electrons transmission under variable temperature fields. The conductivities of the sensor were then investigated. As plotted in Supplementary Fig. 18, the slopes of current-voltage curves were gradually increased as the temperature rises, indicating a positive temperature response of the conductivity. Indeed, the measured resistance was dropped from 4.36 MΩ at 0 °C to 0.093 MΩ at 80 °C, showing excellent temperature sensitivity. Figure 4b presents the variation curves of the normalized resistance change ratio (*R*/*R*_0_) below the starting temperature (T_s_, 24 °C). Notably, *R*/*R*_0_ upsurges sharply first during fast cooling process from 24 °C to target temperatures (0, 3, 10, and 15 °C) and then reaches a steady level within 90 s, next fall back to the starting state and the peak values of *R*/*R*_0_ are consistent with the results from Supplementary Fig. 18, which can be repeated 3 times successfully at each target temperature. In addition, *R*/*R*_0_ demonstrates the opposite trend when the target temperatures exceed T_s_ in Fig. 4c and Supplementary Fig. 19. Briefly, beginning with T_s_, it takes 120 s for *R*/*R*_0_ to decrease dramatically and keep steady, and then recover successfully under cooling process. Meanwhile, the whole process can still be repeated and the minimum *R*/*R*_0_ match the results in Supplementary Fig. 18.

The responses of *R*/*R*_0_ to temperature ranging from 0 to 80 °C fully prove that the printed hydrogel can be suitable for steady temperature sensing. Consequently, the performance of hydrogel thermistor can be evaluated by temperature coefficient of resistance (*TCR*), according to the equation:

1 $${TCR}\,=\,(\triangle R{{ / }}{R}_{0}){{ / }}\triangle T$$

where *T* refers to the temperature. The results were fitted in Fig. 4d and the negative *TCR* were calculated as −5.27% °C^−1^ and −1.11% °C^−1^ at the temperature from 0 to 30 °C and 30 to 80 °C, which surpasses the performance of most polymer composites based temperature sensors. The comparison of composition and properties is summarized in Table S1. Furthermore, similarly data are fitted according to the following equation^43^:

2 $$R\,=\,{R}_{0}{{{{{{\rm{e}}}}}}}^{\frac{B}{T}}$$

where *B* is represented by the materials constant of the thermistor. The resultant *B* is calculated as 4428 K in Supplementary Fig. 20 for the overall temperature range from 0 to 80 °C. In contrast, *TCR* of MXenes unloaded hydrogel thermistor are obtained as −3.83% °C^−1^ and −0.74% °C^−1^ within the same sensing range from Supplementary Fig. 21a, the *B* of the system is fitted to 2011 K from Supplementary Fig. 21b, indicating that the doping of Ti_3_C_2_T_x_ flakes is beneficial to strengthen the sensitivity. Furthermore, Supplementary Fig. 22a demonstrates the hydrogel without glycerol and MXenes performed lower *TCR* of −2.82% °C^−1^ (0 °C to 30 °C) and −0.56% °C^−1^ (30 to 60 °C), the corresponding *B* in Supplementary Fig. 22b declines to 1576 K, which is close to the results from Supplementary Fig. 21b. Note that the hydrogel begins to melt above 60 °C as a result of PVA crystalline domains being disintegrated, meaning that the detecting range of the hydrogel thermistor can be expanded through adding glycerol, but the sensitivity can be hardly affected by glycerol. The hydrogel thermistor without Ti_3_C_2_T_x_ and glycerol exhibits weak temperature response behavior mainly by means of carrier mobility being accelerated through the gradually heated ambiences. Besides, the absence of glycerol results in continual water evaporation of the hydrogel, which can be intensified with temperature rising, leading to a weak temperature sensitivity.

For the results of Fig. 4d and Supplementary Fig. 19, considering the dehydration of hydrogel under heated environment, the solvent content of the two hydrogel systems under 3 heating-cooling cycles mentioned above was measured at each highest target temperature (80 and 60 °C). From Supplementary Fig. 23a, the solvent content of glycerol loaded gel was kept at 89% after 3 cycles at 80 °C, but the system without glycerol presented a severe dehydration at 60 °C (76%). Interestingly, the solvent content of glycerol loaded gel can be rapidly recovered to 99.4% after immersing in glycerol aqueous solution for 120 s owing to strong water absorption ability of glycerol, while it spent nearly 10 h to be recovered at 93% for the gel without glycerol, meaning that the hydrogel with glycerol can be “recharged” like commercial battery through immersing method. Generally, the melting of hydrogel with pristine H_2_O system occurs at temperatures above 60 °C due to the disintegration of PVA microcrystalline domains, which encourages further exploration about the gel stability at 80 °C after adding glycerol. Supplementary Fig. 23b demonstrates the XRD patterns of glycerol loaded gel under various circumstances. Compared with the initial state (at T_s_, i.e., before heating-cooling cycles), the crystallization peak intensity of PVA was obviously increased after 3 cycles at 80 °C with a shortened full width at half maxima (FWHM), but the peak intensity and FWHM recovered to initial state after “recharging” the hydrogel as a result of the dehydration and rehydration process, which means that PVA microcrystalline domains can be preserved under high temperature after adding glycerol. Hence, the adding of glycerol promotes the hydrogen bonding between the solvents and solutes, which not only locks the water in the system, but also enhances the chains interaction between PU and PVA, ensuring the water retention during heating process and the stability of PVA crystalline domains at high temperature. Consequently, the dissolution of the hydrogel at high temperature can be prevented, broadening the sensing range of the thermistor.

In addition, the conductivity of gel can be inevitably affected during dehydration, from Supplementary Fig. 24, the resistance of glycerol loaded gel was slightly increased from 1.97 to 2.76 MΩ at the end of the third heating-cooling cycle, then dropped back to 2.0 MΩ after being “recharged”. Furthermore, thermal sensing behaviors of “recharged” hydrogel was measured from 0 to 80 °C under T_s_ of 24 °C. Similarly, the resistance of the “recharged” gel can be heightened when the target temperature is lower than T_s_ (Supplementary Fig. 25a), but decreased when the temperature surpasses T_s_ (Supplementary Fig. 25b). the peak values of *R*/*R*_0_ dropped apparently from 2.28 at 0 °C to 0.13 at 80 °C, and 3 heating-cooling cycles were achieved for each target temperature. As a result, *TCR* values of “recharged” gel were fitted in Supplementary Fig. 26a, delivering that −5.15% °C^−1^ (0 to 30 °C) and −1.03% °C^−1^ (30 to 80 °C), which is strongly close to the results of Fig. 4d, and the resultant *B* value was calculated as 3524 K (Supplementary Fig. 26b). From the results analyzed above, the printed hydrogel presents superior temperature sensing performance, even if slight dehydration occurs at high temperature, the “recharging” process enables the gel being reused stably.

For further clarification, the effects of Ti_3_C_2_T_x_ flakes on thermal conductivity (*λ*) and thermal diffusion (*α*) of thermistors are explored. As shown in Supplementary Fig. 27a, b, both the *λ* and *α* are enhanced from 0.37 W ∙ (m ∙ K)^−1^ to 0.39 W ∙ (m ∙ K)^−1^ and 0.13 mm^2^ ∙ s^−1^ to 0.15 mm^2^ ∙ s^−1^ with MXenes loaded. The thermal conductivity is closely related to chemical bonds vibration, hence temperature-dependent Raman spectra was also performed to investigated the vibration modes of the hydrogel composite. The results of Ti_3_C_2_T_x_ flakes at variable temperature from 0 to 80 °C are shown in Supplementary Fig. 28a, strong peaks near 202 cm^−1^ are attributed to a group vibration of carbon and titanium layers, corresponding to thermal-induced in-plane (E_g_ modes) and out-of-plane (A_1g_ modes) vibration of Ti and C atoms. The fitted and normalized data of (202) peaks were plotted in Fig. 4e,  f, it is clearly that the peak intensities are not affected by temperature, but a shift from 196 to 191.9 cm^−1^ of peak positions (Supplementary Fig. 29a) can be noticed, which will be discussed later. For Ti_3_C_2_T_x_ bonded hydrogel, the full Raman spectra is shown in Supplementary Fig. 28b, the distinctive peak intensities near 2921 cm^−1^ are attributed to thermal-induced stretch vibration of methylene groups (*ν* C–H) on PVA and PU chains, while Ti_3_C_2_T_x_ presents relatively weak vibration as a result of less loading (0.25 wt%). From the results of fitted and normalized data (Fig. 4g, h), the peak intensities of methylene groups vibration are weakened, but the peak positions shift toward higher from 2913 to 2926 cm^−1^ frequency (Supplementary Fig. 29b) with temperature increase. The observed variation of Raman peak positions and peak intensities with temperature can be attributed to thermal expansion and harmonic potential constant related to the anharmonicity.

Based on the results discussed above, the response mechanism of thermal-induced tunneling effect can be refined as follows. Briefly, in the hydrogel without MXenes, the heat transfer is dominated by the vibrations of PVA crystalline domains and movement of polymer chains. Whereas in the hydrogel with MXenes loaded, the MXenes network with high thermal conductivity will ensure the rapid temperature response. Meanwhile, thermal disturbance caused by the raised temperature provides sufficient energy for an increasing number of electrons to achieve the transition, as depicted in Fig. 4i. Specifically, the effect of temperature on the conductivity of MXene mainly depends on the electronic properties and microscopic configuration of Ti_3_C_2_T_x_. According to the band structure analysis, bare Ti_3_C_2_ exhibits strong metallicity, while surface functionalized Ti_3_C_2_T_x_ presents narrow bandgap semiconducting properties^44,45^. Presence of T groups (–F and –OH) results in the transformation of metallic Ti_3_C_2_ into Ti_3_C_2_T_x_ with bandgaps of 0.05 and 0.1 eV, respectively. In addition, the T groups bonded with Ti atoms are located in hollow sites (type I) above adjacent carbon atoms, exhibiting semiconductive properties^46,47^. If oriented on the topmost of the C atoms, the materials are metallic (type II). Generally, Type I is relatively more energetically stable for Ti_3_C_2_T_x_. Based on the narrow bandgap semiconductive behavior, heat endows electrons with sufficient energy to achieve transitions as well as boost carrier concentration. Therefore, the resultant conductivity of MXene can be heightened with the increasing temperature. In accordance with the tunneling effect, the effective and efficient electrons transmission can be facilitated by the excellent conductivity of Ti_3_C_2_T_x_, hence promoting the electron transmission efficiency.

Owing to the superior thermal conductivity of the hydrogel, the heat from human hand can be quickly dissipated through the printed sensor when attached to skin, as recorded in the infra (IR) images before and being attached for1h from Supplementary Fig. 30. Additionally, owing to the superior and stable thermal sensing performance, the printed thermistor can be used for detecting surface temperature of objects in real time. The thermistor was attached to the bottom of cup outside wall with 40 mL cool water (24 °C) contained, then 15 mL hot water (90 °C) was successively added for 5 times. The increasing temperature was recorded through *R*/*R*_0_ change fitted in Supplementary Fig. 31a, R/*R*_0_ decreased after adding hot water each time, and the obtained temperature from R/R_0_ change was calculated according to *TCR* results (Fig. 4d). Compared to temperature measured by commercial IR camera (Supplementary Fig. 31b), the printed thermistor can precisely detect the temperature of objects.

### Application scenario simulation of printed thermistor

**Fig. 5:**
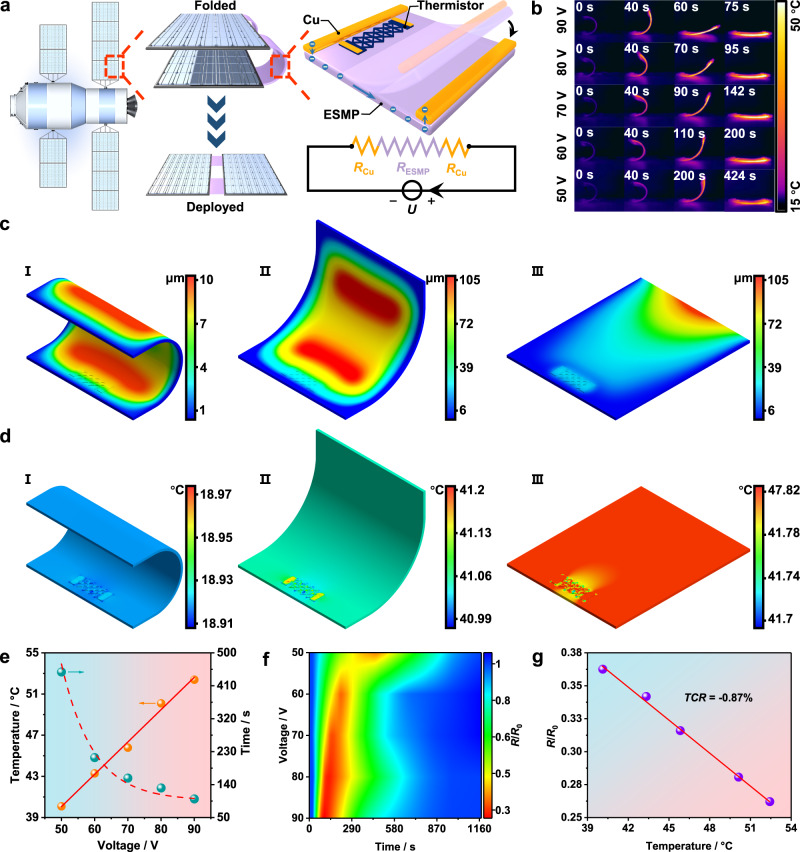
Construction and operation of temperature monitoring system. **a** Schematic illustration of solar array deployment process and electro-induced shape memory polymer composites (ESMP) device design. **b** Thermal images of system deployment process under different voltages. Finite element analysis of (**c**) total instantaneous deformation and (**d**) temperature distribution at different stages. **e** Functional relationship among final deployment temperature, time and applied voltage. **f** Resistance changes of the thermistor attached on ESMP and (**g**) the resultant Temperature coefficient of resistance (*TCR*) value.

The printed hydrogel thermistor with satisfactory sensitivity performs remarkably for the application of temperature indication of solar array hinge, which can be constructed by electro-induced shape memory polymer composites (ESMP)^48,49^. Typically, as demonstrated in Fig. 5a, the solar array deployment requires the unfolding of ESMP hinges activated by Joule heat, and the printed thermistor can be attached on ESMP surface, both of the thermistor and ESMP were connected by copper electrodes, respectively. After electrifying the system, electrons pass through the series circuit consisting of ESMP and copper for providing Joule heat, which can be detected by hydrogel thermistor.

Figure 5b and Supplementary Movie 2 record the deployment processes of the system under variable voltages by infrared thermal camera. The temperature of ESMP and thermistor rise gradually and the final deployment time (*t*_r_) drops from 424 s at 50 V to 75 s at 90 V. Moreover, finite element models were constructed to investigate the thermomechanical behavior of the system under 70 V voltage. Specifically, on the one hand, Fig. 5c presents the total instantaneous deformation of ESMP under different deployment stages. At initial state (the moment of system power-on), weak deformation caused by less Joule heat is distributed at both sides of ESMP (Fig. 5c, I), which can be strengthened with the accumulation of Joule heat at semi-deployed state (Fig. 5c, II), but the deformation is concentrated on one side of ESMP end state when the deployment is complete (Fig. 5c, III). On the other hand, temperature and heat flux distribution of hydrogel thermistor is presented in Fig. 5d and Supplementary Fig. 32, respectively. Both results demonstrate a continual rise from the initial state to end state, meaning that effective heat conduction is formed between ESMP and hydrogel thermistor. Based on the theoretical results, the actual surface temperature distributions at end state are recorded in Supplementary Fig. 33 firstly, the relationship among *t*_r_, final deployment temperature (*T*_r_) and applied voltage is revealed in Fig. 5e, note that *T*_r_ is defined as the surface temperature near the hydrogel sensor of ESMP detected by the infrared camera, which presents a linear relationship with applied voltages in a range of 50–90 V. Furthermore, the resistance responses of the hydrogel thermistor during deployment at variable applied voltages is presented as color map in Fig. 5f. The mounting voltages accelerates the boost of ESMP surface temperature, resulting in a progressively rapid decrease in *R*/*R*_0_. After powering off, *R*/*R*_0_ gradually return to original value as ESMP cools naturally. Finally, the corresponding *TCR* was calculated as −0.87% °C^−1^ after fitting the *R*/*R*_0_ at the ending state with the actual temperature, which is close to the results from Fig. 4d, presenting that the printed thermistor can linearly reflect the actual temperature change.

## Discussion

In summary, Ti_3_C_2_T_x_ bonded polymer hydrogel was prepared through a facile 3D printing strategy of DIW technology and solvent replacement method. During the gelation, PVA crystalline domains were generated, and an abundance of hydrogen bonds among solvent, matrices and fillers were formed. Owing to the relative slippage of Ti_3_C_2_T_x_ flakes and the distinctive printing structure, stretchable resistive sensor based on the printed hydrogel performed noticeable strain responses with the maximum GF of 5.7 within 191% strain, response time of 240 s and stability over 5000 cycles, which presented timely feedback of human movement detection. Further, on the basis of water retention of glycerol, and the synergy between temperature and tunneling effect, the as printed hydrogel demonstrates terrific temperature sensitive behaviors with *TCR* of −5.27% °C^−1^ (0 °C–30 °C) and −0.84% °C^−1^ (40 °C–80 °C). Additionally, an electro-induced shape memory system with printed hydrogel sensor was successfully constructed, and the sensor precisely indicated the shape recovery temperature.

## Methods

### Materials

All chemical regents employed in this research are available commercially and were used as received without further purification. Ti_3_AlC_2_ powders was purchased from Jilin 11 technology Co., Ltd. PVA (98-99 mol% alcoholysis, *M*_w_ ≈ 7.5 × 10^4^ g/mol), LiF (AR, 99%), glycerol (GC, ≥99.5%) and L-ascorbic acid (ACS, ≥99%) were supplied by Shanghai Aladdin Biochemical Technology Co., Ltd. Hydrochloric acid (HCl, GR, 36–38 wt%) was provided by Sinopharm Chemical Reagent Co., Ltd. PU (50% solid content) was obtained from Guangzhou Hensic New Materia Co., Ltd. Deionized water was used during the course of entire experiment.

### Preparation of Ti_3_C_2_T_x_ flakes

The etching of Ti_3_AlC_2_ was followed by the minimally intensive layer delamination method. Specifically, 0.8 g LiF were carefully mixed with 10 ml 9 M HCl solution, which was kept stirring within Teflon agitator for 10 min. Next, 0.5 g Ti_3_AlC_2_ powders was gradually added into the blend mentioned above over 10 min, later the black mixture was stirred continually at 35 °C for 24 h. The obtained suspension was washed with deionized water repeatedly until pH exceeds 6, then the centrifugation of 1414 × *g* with 2 min was constantly carried out for collecting multilayered Ti_3_C_2_T_x_. The collected supernatant was sonicated in ultrasonic bath for 40 min under the protection of Ar atmosphere. Delaminated Ti_3_C_2_T_x_ flakes were acquired through further centrifugation for 1 h at 1414 × *g* followed by lyophilization, and the final products were stored in Ar atmosphere.

### Preparation of hydrogel precursor

Typically, 9 mg (0.25 wt%) Ti_3_C_2_T_x_ flakes were dissolved in 1 g deionized water (DI), 1.5 g glycerol (60 wt% of DI/glycerol mixture) was injected gradually into MXenes solution after sonication of 10 min. Next, 0.715 g (10 wt%) waterborne PU was added drop by drop into the mixture for achieving homogenous solution with the help of vigorous stirring. After blending 0.35 g (10 wt%) PVA, the whole system was sealed under Ar atmosphere and then heated at 90 °C for 1.5 h to obtain viscous hybrid precursor. The control samples of different weight ratios of MXenes, glycerol and PU were prepared followed by same procedure.

### 3D printing of Ti_3_C_2_T_x_ loaded hydrogel

For DIW 3D printing, microelectronic printer from Prtronic was adopted in this research. Typically, dispensing syringe was fixed on the supports after 0.25 wt% Ti_3_C_2_T_x_ loaded precursor solution was transferred into that, and the top of the syringe was connected with intake hose. After inserting the supports on the metallic tray, a PET film was absorbed firmly on the printing platform through air pumping. The designed printing patterns were fabricated through corresponding software Bits Assembler. The viscous precursor was extruded on the PET film following the designed paths under the printing pressure of 80 KPa, the printing speed and building height were controlled at 1 mm/s and 23.6 mm respectively. Finally, the printed precursors were preserved at −20 °C for 72 h to achieve gelation and soaked in 0.25 wt% L-ascorbic acid glycerol aqueous solution, same with the formulation of precursor preparation.

### General characterization

The morphology and structure of Ti_3_AlC_2_ powders, Ti_3_C_2_T_x_ flakes and hydrogel were observed with the aid of FESEM (FEI, Verios G4), hydrogels were immersed in deionized water to remove glycerol before brittle ruptured with the aid of liquid nitrogen, for ensuring thorough lyophilization. The thickness of Ti_3_C_2_T_x_ flakes was measured through atomic force microscope (AFM, Bruker, Dimension Icon). Crystalline structure of Ti_3_AlC_2_, Ti_3_C_2_T_x_ and hydrogel were identified by XRD (Bruker, D8 Discover, Cu Kα, 2*θ* scan range of 5°–80°), and the chemical composition of Ti_3_C_2_T_x_ and hydrogel was investigated by X-ray photoelectron spectroscopy (XPS, Kratos AXIS Ultra DLD, Al Kα) and Fourier Transform Infrared spectrometer (FTIR), respectively. Solvent content was measured through weighing method. Mechanical tests of hydrogel samples were determined via electronic universal testing machine (INSTRON, 3344), all samples were casted as standard dumbbell-shaped specimens of 75 mm length, 12.5 mm wideness, and 1 mm thickness with same gelation procedure for stretching tests, the samples of 15 mm diameter and 10 mm thickness were used for compression-recovery tests. Dynamic mechanical analysis (NETZSCH, DMA 242E) tests were performed at 1 Hz within −150 °C to 15 °C for stretching samples of 30 mm length, 5 mm wideness, and 1 mm thickness. The standard dumbbell-shaped specimens were used directly as sensing devices with each end being connected by copper wires for investigation about the effect of Ti_3_C_2_T_x_ loadings on strain sensing, and the gauge range was set as 30 mm. For surface temperature control, a temperature controlling system including semiconductor chilling plates and planar heater was applied. Current, voltage and resistance change were recorded by digital source meter (Kethley 2450) during strain and temperature sensing. Infrared thermal images were collected through thermal camera (FLIR E5XT). Raman spectra were investigated by Raman spectrometer (WITec, Alpha300R) under 488 nm laser excitation. Thermal conductivity and thermal diffusivity values were acquired through thermal constant analyze equipment (Hot Disk, TPS2200) with the atmospheric pressure and the samples dimension of 20 mm × 20 mm × 2 mm. *T*_r_ monitoring system construction: thin copper sheets were attached to both ends of ESMP and connected to the voltage regulator, polymethylmethacrylate (PMMA) insulating plates were fixed on the copper sheets for ensuring insulation. Then the thermal conductive tape was sandwiched by ESMP and copper wires connected printed sensor to prevent short circuit, the printed sensor was connected to obtain real-time resistance during entire recoveries. All human body related experiments of on-skin devices on a volunteer were performed in compliance with the relevant laws and institutional guidelines and under approval from Ethics Committee of Northwestern Polytechnical University (Project number: 202102010). All human subjects gave written and informed consent before participation in the experiments.

## Supplementary information

Supplementary Information

Peer Review File

Description of Additional Supplementary Files

Supplementary Movie 1

Supplementary Movie 2

## Data Availability

Source data are provided with this paper.

## Source data

Source Data
