# Supplementary material for: Approaching intrinsic dynamics of MXenes hybrid hydrogel for 3D printed multimodal intelligent devices with ultrahigh superelasticity and temperature sensitivity
Source: Nat Commun. 2022 Jun 14;13:3420. doi: 10.1038/s41467-022-31051-7 (PMC9197829; doi:10.1038/s41467-022-31051-7)
Supplement: Supplementary file 1 — Supplementary Information [file 41467_2022_31051_MOESM1_ESM.pdf]

Supplementary Information for

Approaching Intrinsic Dynamics of MXenes Hybrid Hydrogel for 3D Printed Multimodal Intelligent Devices with Ultrahigh Superelasticity and Temperature sensitivity

Haodong Liu[1,7], Chengfeng Du[2,7], Liling Liao[3,7], Hongjian Zhang[1], Haiqing Zhou[3], Weichang Zhou[3], Tianning Ren[4], Zhicheng Sun[1], Yufei Lu[1], Zhentao Nie[1], Feng Xu[1], Jixin Zhu,[5,6]* Wei Huang[1,6]*

[1]Frontiers Science Center for Flexible Electronics (FSCFE), Xi'an Institute of Flexible Electronics (IFE), Xi'an Institute of Biomedical Materials & Engineering, Northwestern Polytechnical University (NPU), 127 West Youyi Road, Xi'an, 710072, P. R. China.

[2]State Key Laboratory of Solidification Processing, Center of Advanced Lubrication and Seal Materials, Northwestern Polytechnical University (NPU), 127 West Youyi Road, Xi'an, 710072, PR China.

[3]Key Laboratory of Low-Dimensional Quantum Structures and Quantum Control of Ministry of Education, Key Laboratory for Matter Microstructure and Function of Hunan Province, Department of Physics and Synergetic Innovation Center for Quantum Effects and Applications, Hunan Normal University, 36 Lushan Road, Changsha, 410081, PR China.

[4]School of Chemistry and Chemical Engineering, Northwestern Polytechnical University (NPU), 127 West Youyi Road, Xi'an, 710072, P. R. China.

[5]State Key Laboratory of Fire Science, University of Science and Technology of China, 443 Huangshan Road, Hefei, 230027, P. R. China.

[6]Institute of Advanced Materials (IAM), Key Laboratory of Institute of Advanced Materials, Nanjing Tech University (NanjingTech), 30 South Puzhu Road, Nanjing, 211816, P. R. China.

[7]These authors contributed equally: Haodong Liu, Chengfeng Du, Liling Liao.

*E-mail: zhujixin@ustc.edu.cn; iamwhuang@njtech.edu.cn

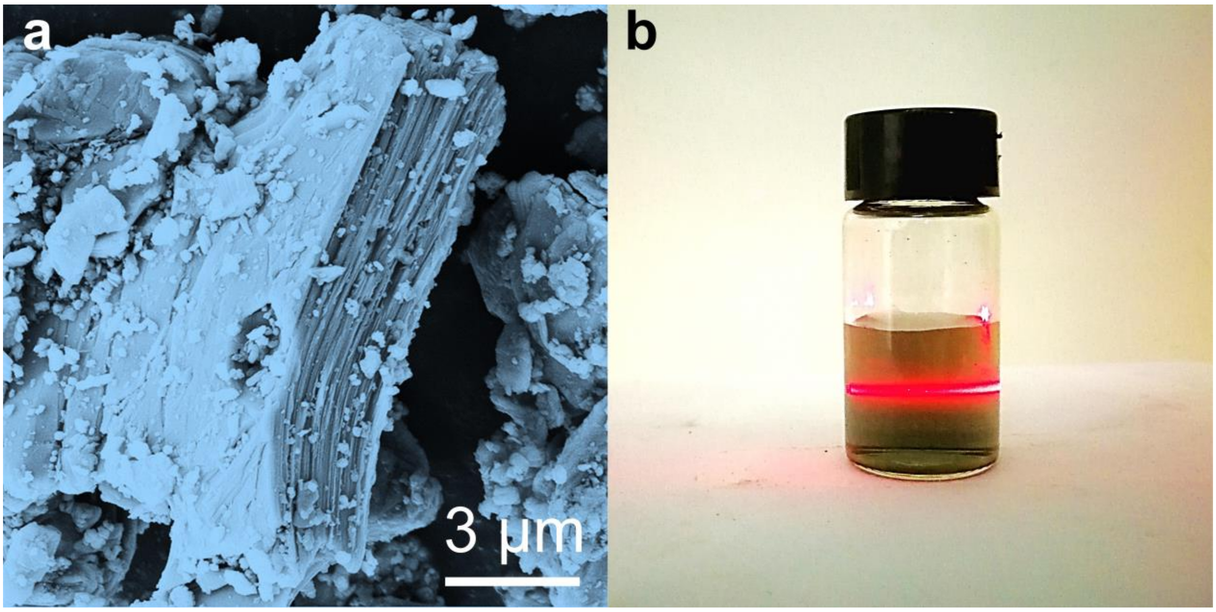

Supplementary Fig. 1 **a** Scanning electron microscope (SEM) morphology of Ti_3AlC_2 powders and **b** Optical photos of Tyndall effect for transition metal carbides (MXenes) suspension.

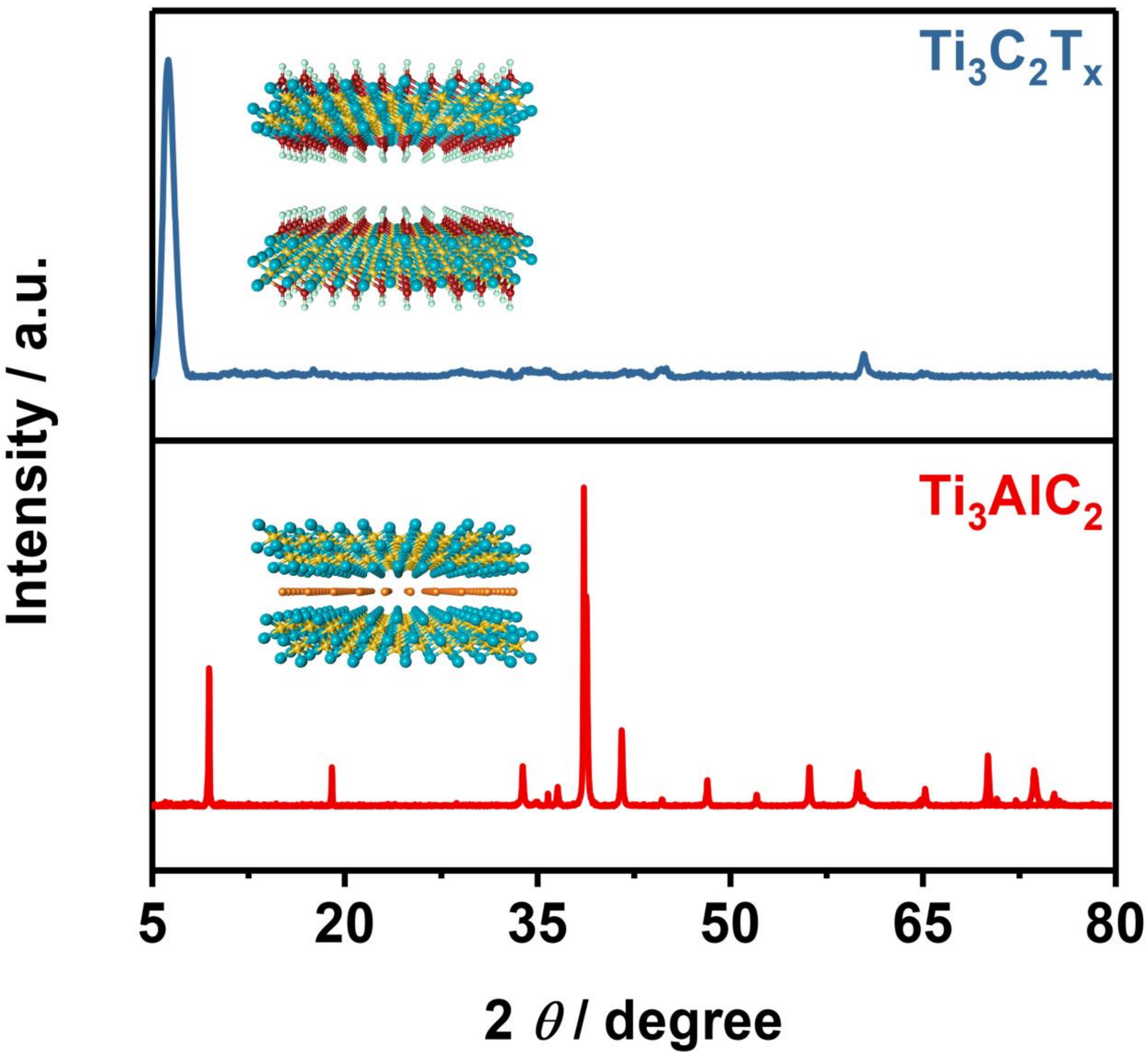

Supplementary Fig. 2 X-ray diffraction (XRD) patterns for Ti_3AlC_2 and the obtained $Ti_3C_2T_x$.

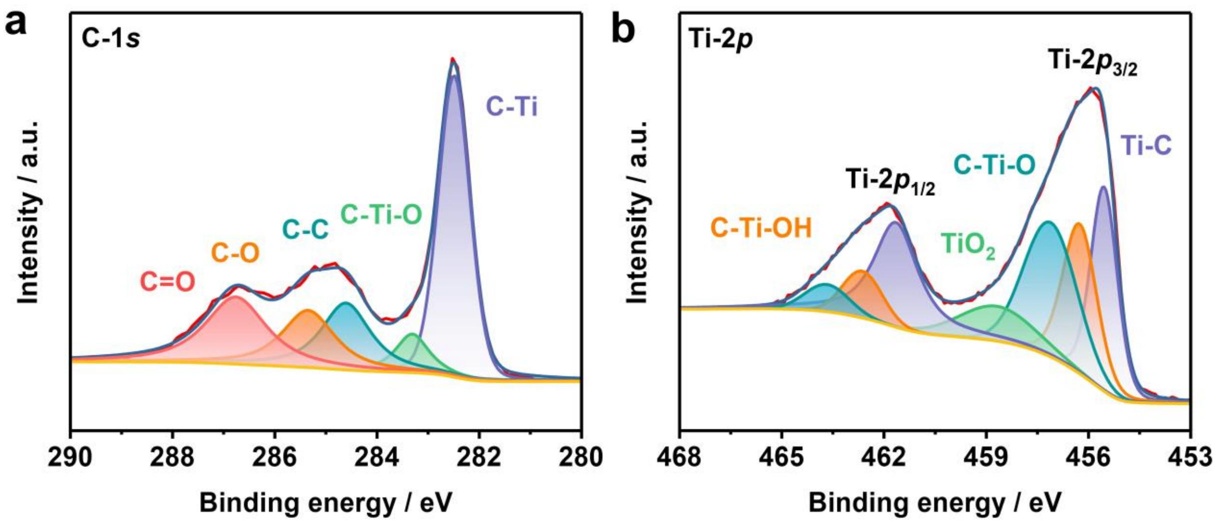

Supplementary Fig. 3 X-ray photoelectron spectroscopy (XPS) fittings for classic **a** C−1*s* and **b** Ti −2*p* of $Ti_3C_2T_x$ flakes.

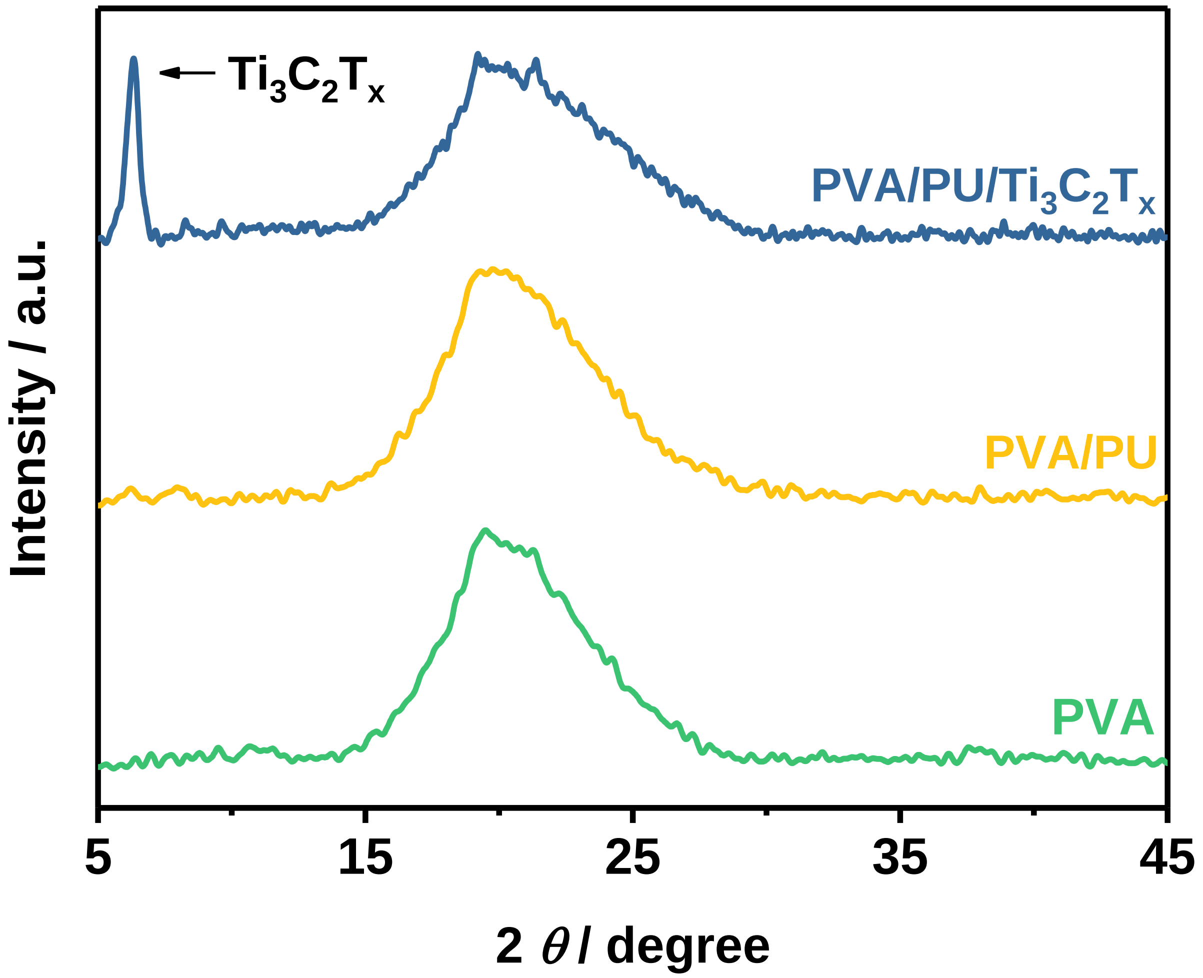

Supplementary Fig. 4 X-ray diffraction (XRD) patterns of glycerol loaded hydrogel with different components.

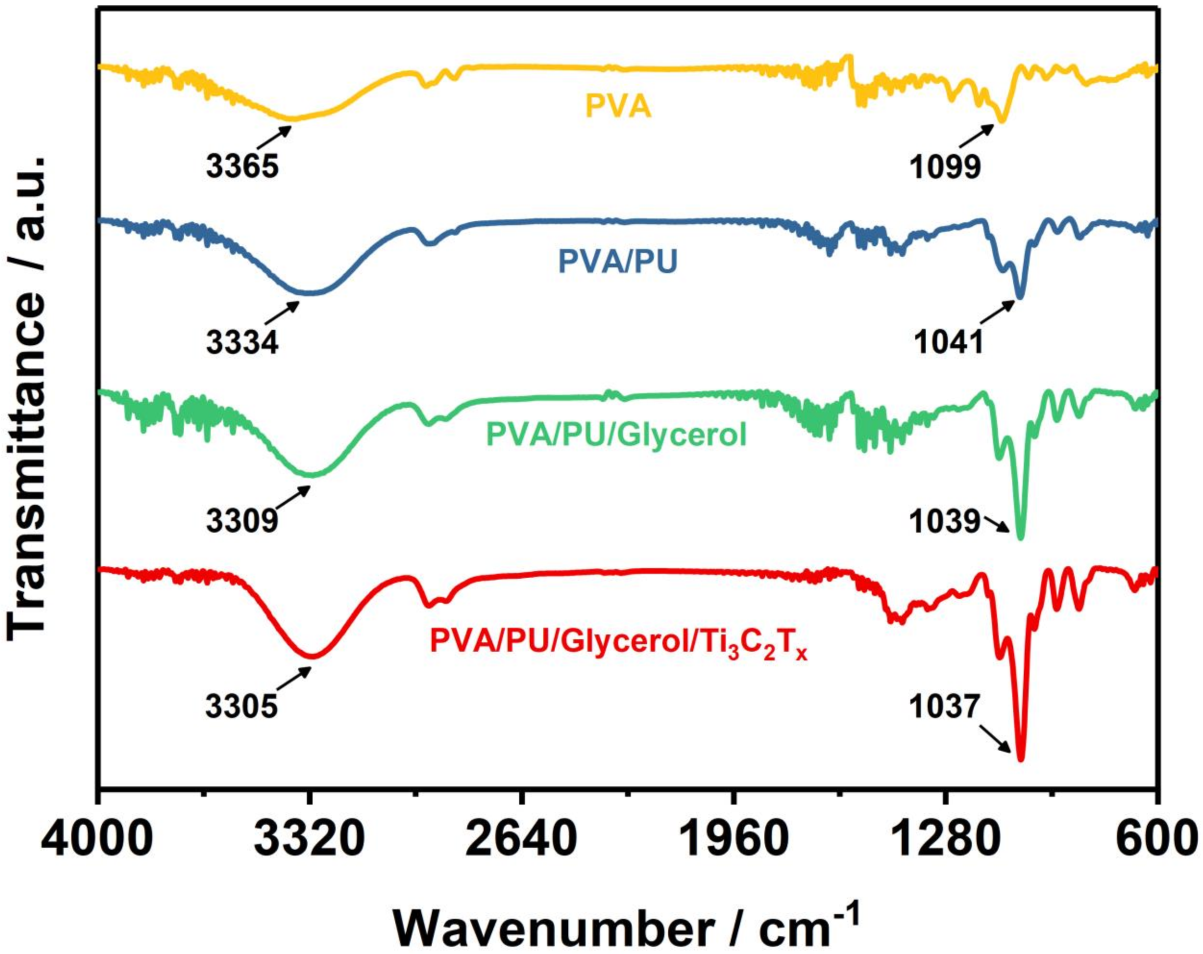

Supplementary Fig. 5 Fourier transform infrared spectroscopy (FTIR) spectrum of hydrogel with various compositions.

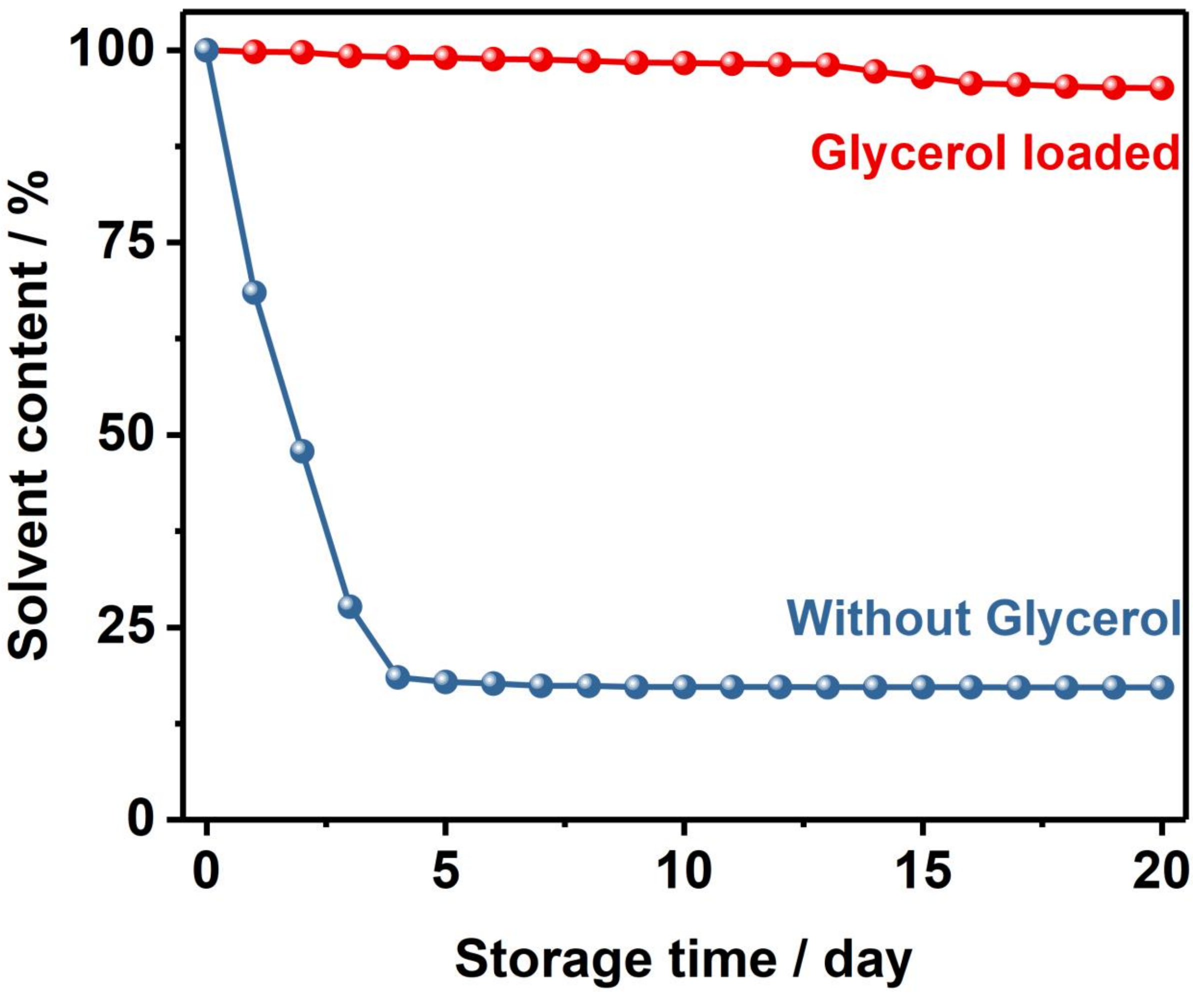

Supplementary Fig. 6 The effect of glycerol on the solvent content (water retention) of hydrogel.

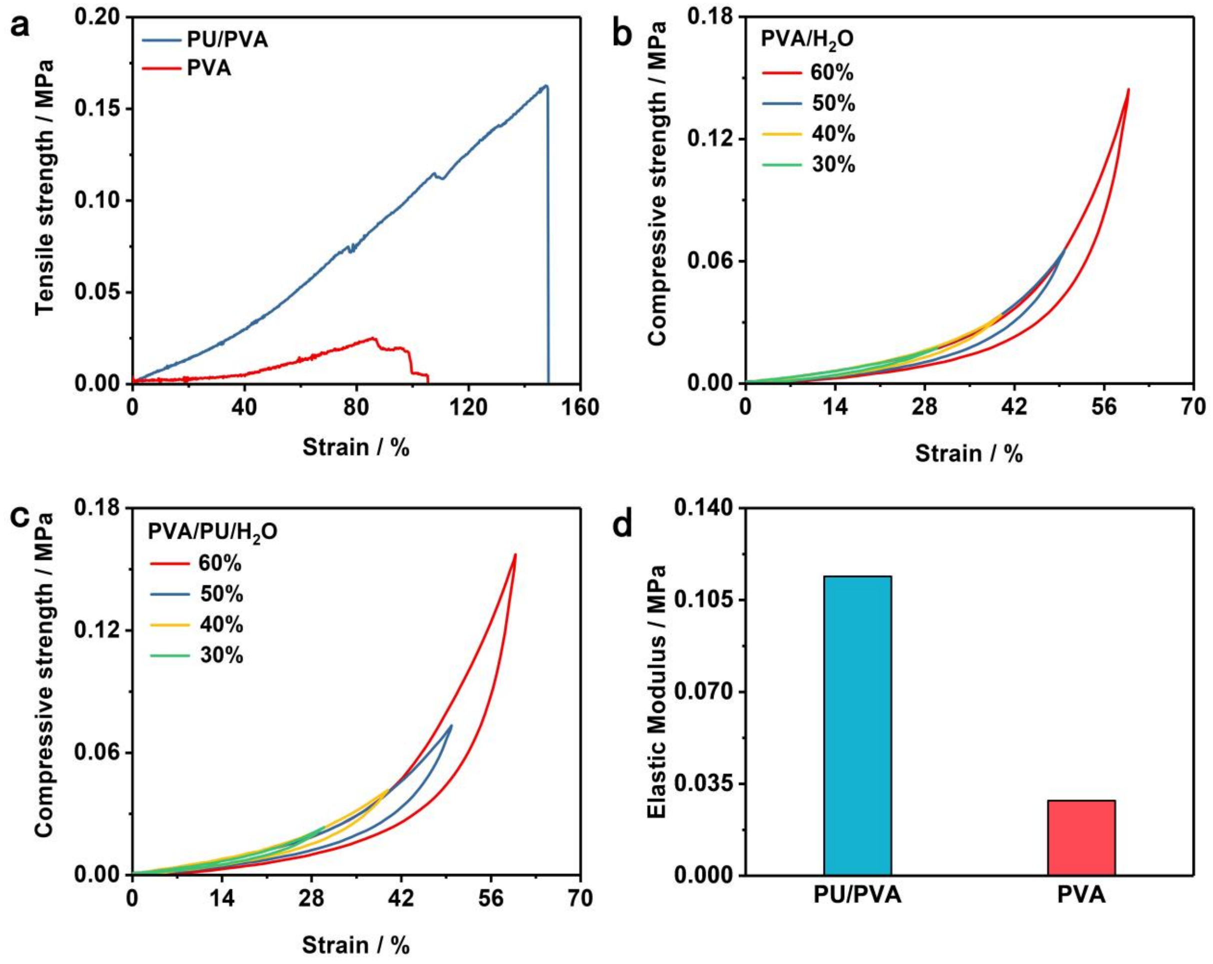

Supplementary Fig. 7 a Tensile strength-strain curves of pristine polyvinyl alcohol/H_2O (PVA/H_2O) hydrogel and polyurethane/polyvinyl alcohol (PU/PVA) hydrogel, the compression-recovery curves of **b** PVA/H_2O hydrogel and **c** PU/PVA hydrogel, **d** elastic modulus of pristine PVA/H_2O hydrogel and PU/PVA hydrogel composites.

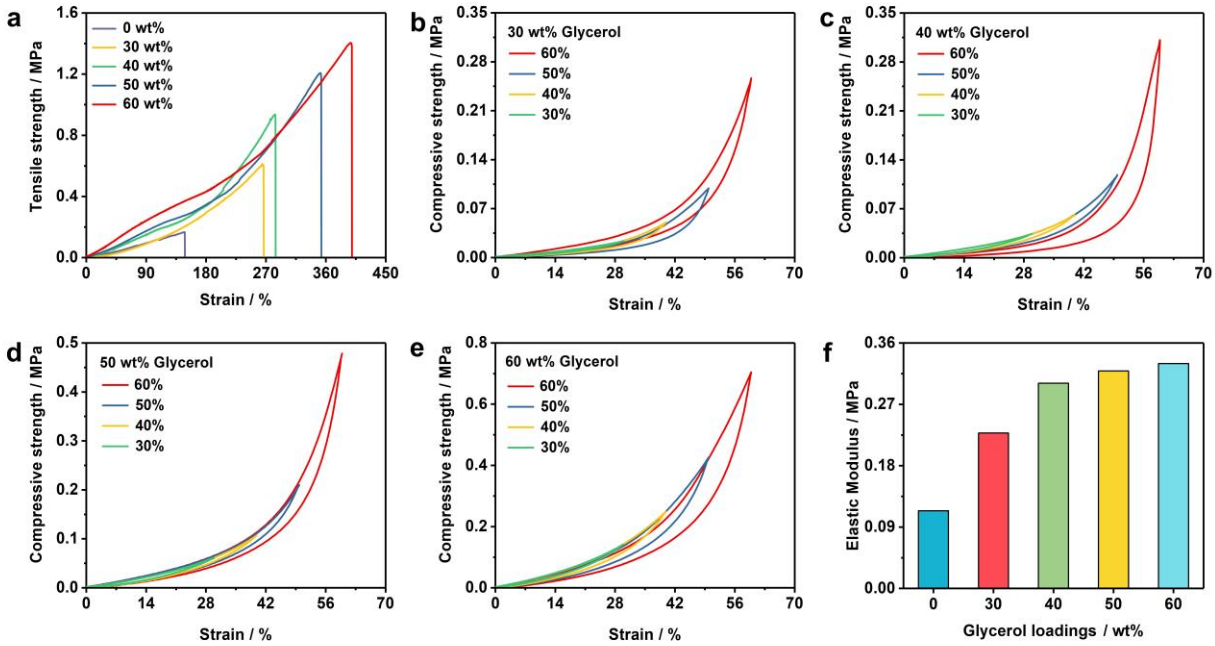

Supplementary Fig. 8 The effect of glycerol loading on **a** tensile strength of hydrogel and **b**−**e** compression-recovery performance, **f** elastic modulus of polyurethane/polyvinyl alcohol (PU/PVA) hydrogel with different glycerol loadings.

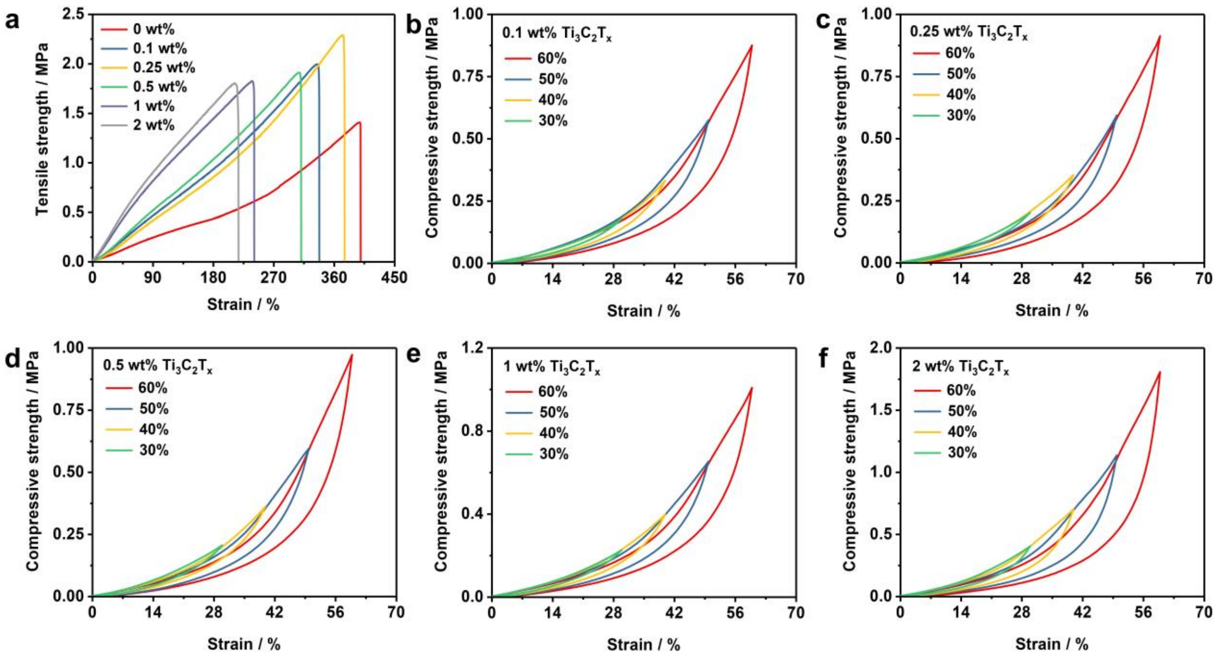

Supplementary Fig. 9 The effect of $Ti_3C_2T_x$ on **a** tensile strength and **b**−**f** compression-recovery performance of polyvinyl alcohol (PVA) hydrogel with polyurethane (PU) and glycerol.

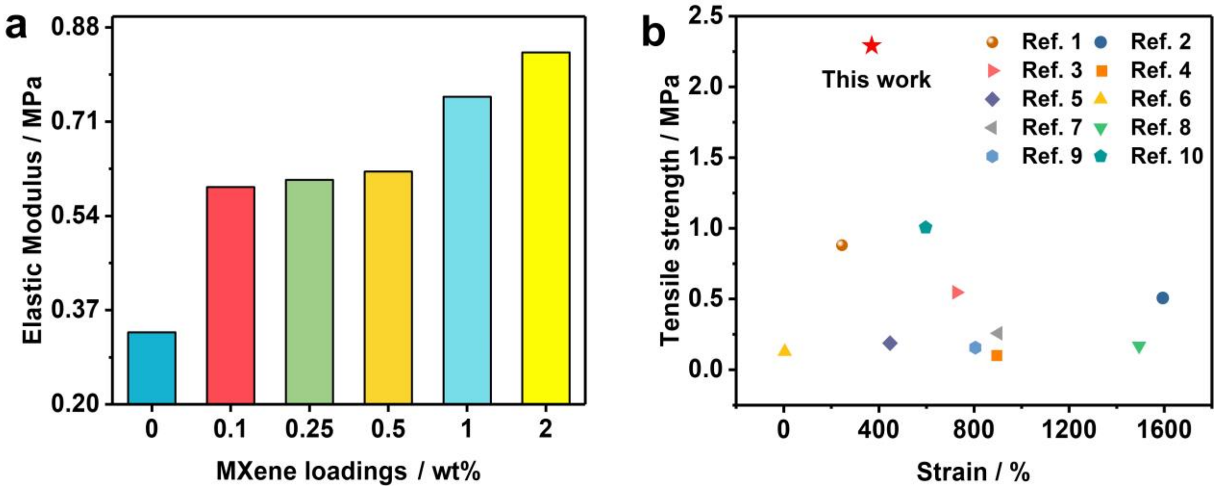

Supplementary Fig. 10 **a** The effect of $Ti_3C_2T_x$ on elastic modulus of polyvinyl alcohol (PVA) hydrogel with polyurethane (PU) and glycerol, **b** the comparison of tensile strength with reported MXene contained hydrogel[1-10].

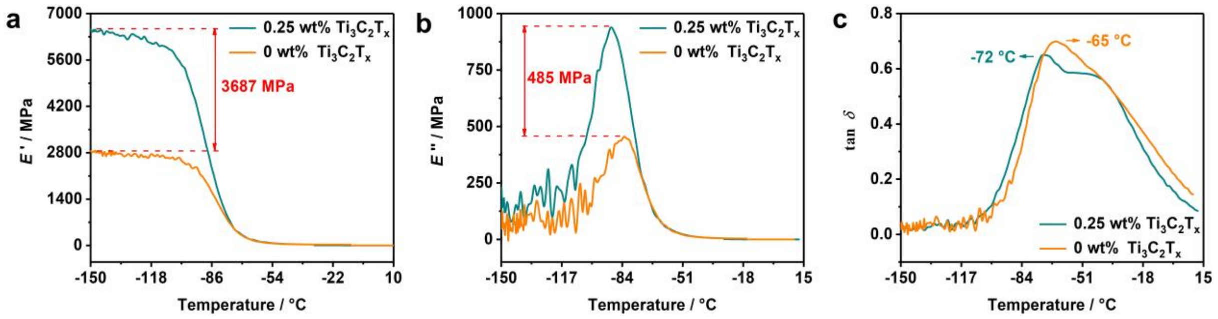

Supplementary Fig. 11 a The effect of $Ti_3C_2T_x$ on storage modulus and **b** loss modulus of polyvinyl alcohol (PVA) hydrogel with polyurethane (PU) and glycerol, **c** the glass transition temperature (T_g) of the two hydrogel samples.

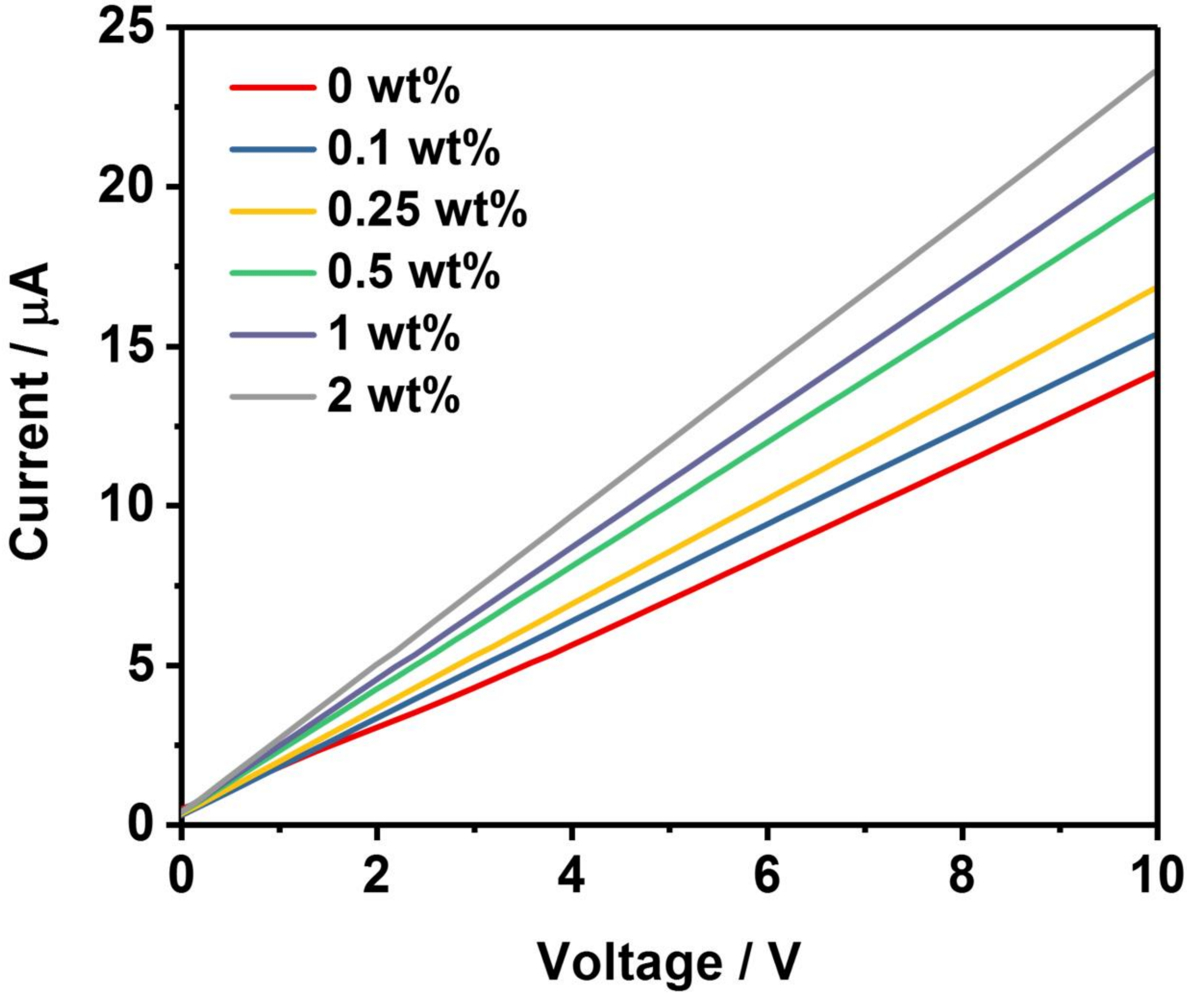

Supplementary Fig. 12 The conductivities of dumbbell-shaped hydrogel specimens with various $Ti_3C_2T_x$ loadings.

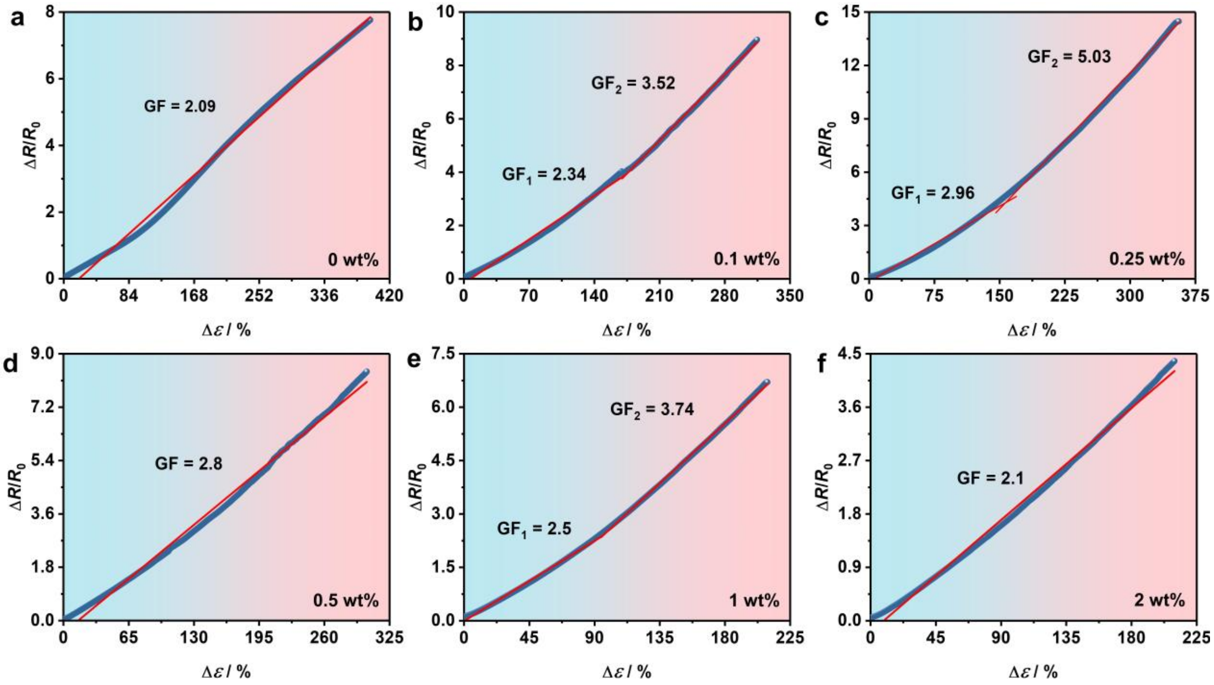

Supplementary Fig. 13 Gauge factor (GF) of dumbbell-shaped hydrogel specimens with various $Ti_3C_2T_x$ loadings. **a** GF of hydrogel with 0 wt% $Ti_3C_2T_x$, **b** GF of hydrogel with 0.1 wt% $Ti_3C_2T_x$, **c** GF of hydrogel with 0.25 wt% $Ti_3C_2T_x$, **d** GF of hydrogel with 0.5 wt% $Ti_3C_2T_x$, **e** GF of hydrogel with 1 wt% $Ti_3C_2T_x$, **f** GF of hydrogel with 2 wt% $Ti_3C_2T_x$.

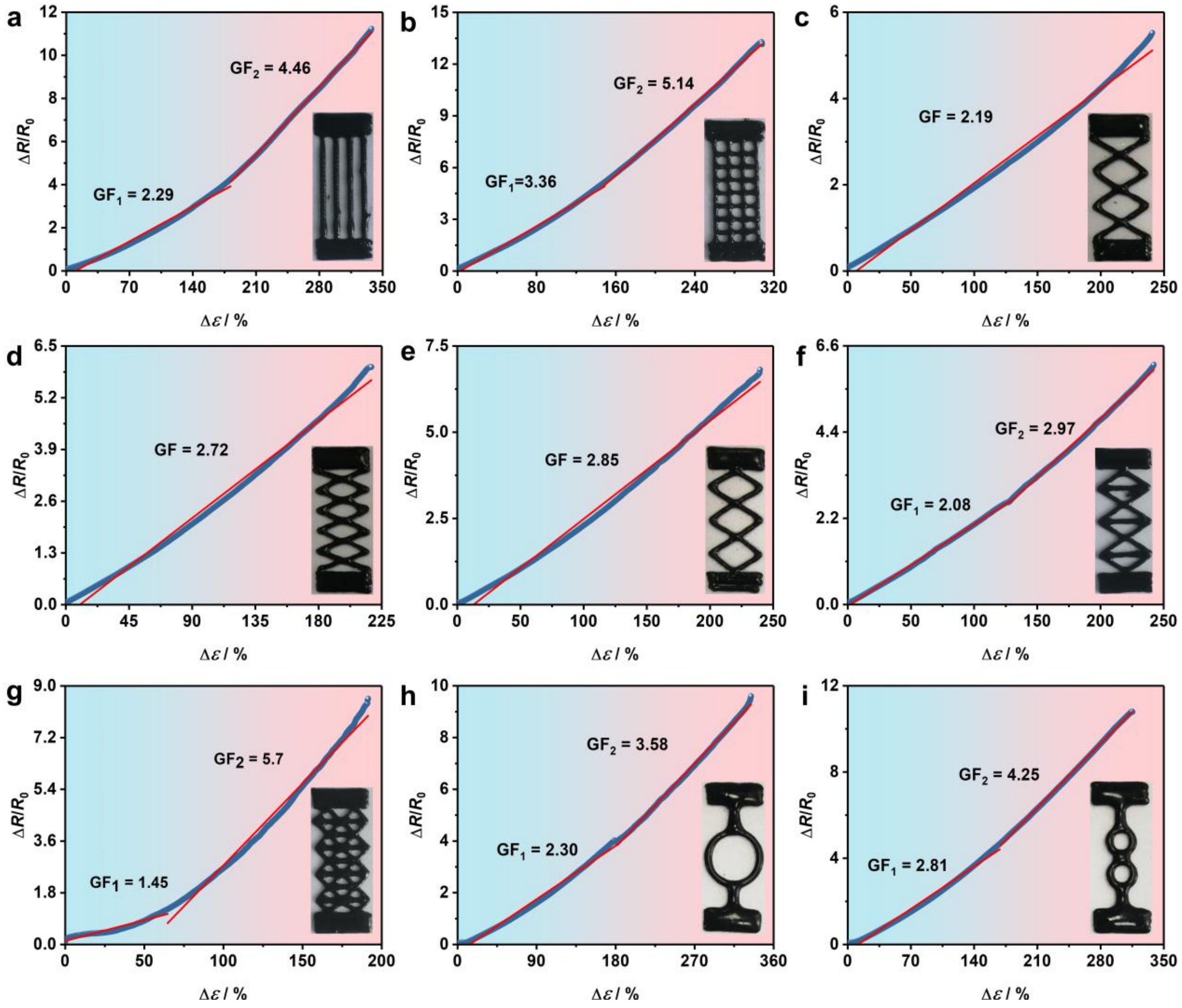

Supplementary Fig. 14 Gauge factor (GF) of different printed patterns with 0.25% $Ti_3C_2T_x$ loading. **a** GF of line patterned device, **b** GF of square patterned device, **c** GF of double-rhombus patterned device, **d** GF of quadruple-rhombus patterned device, **e** GF of triple-rhombus patterned device, **f** GF of cross-beamed rhombus patterned device, **g** GF of multi triangle patterned device, **h** GF of round patterned device, **i** GF of double-round patterned device.

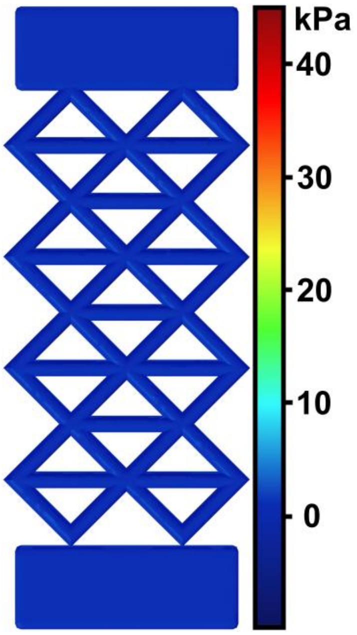

Supplementary Fig. 15 Stress-strain distribution simulation model of initial device.

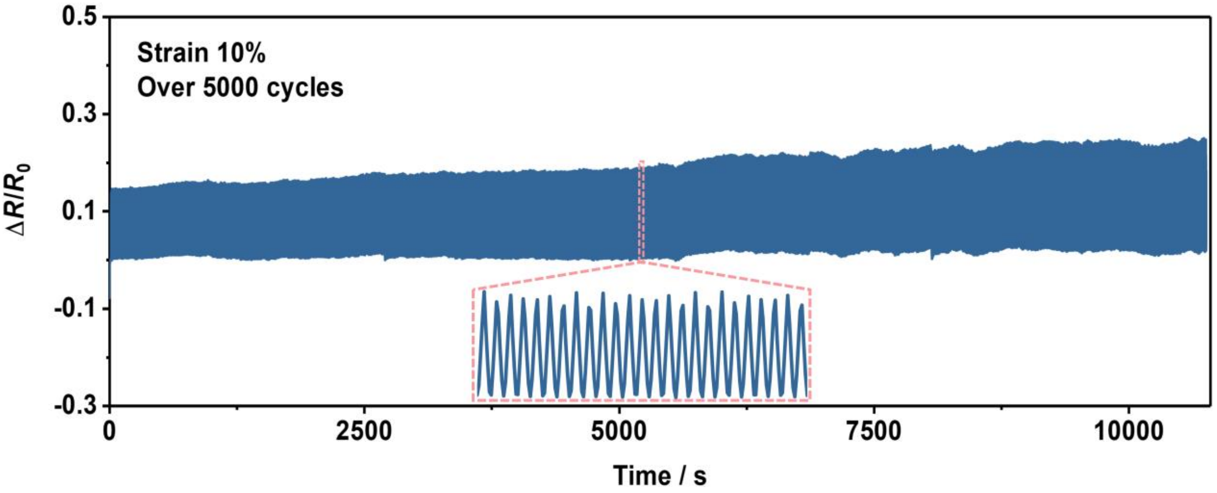

Supplementary Fig. 16 Durability of the sensor under 10% strain over 5000 cycles.

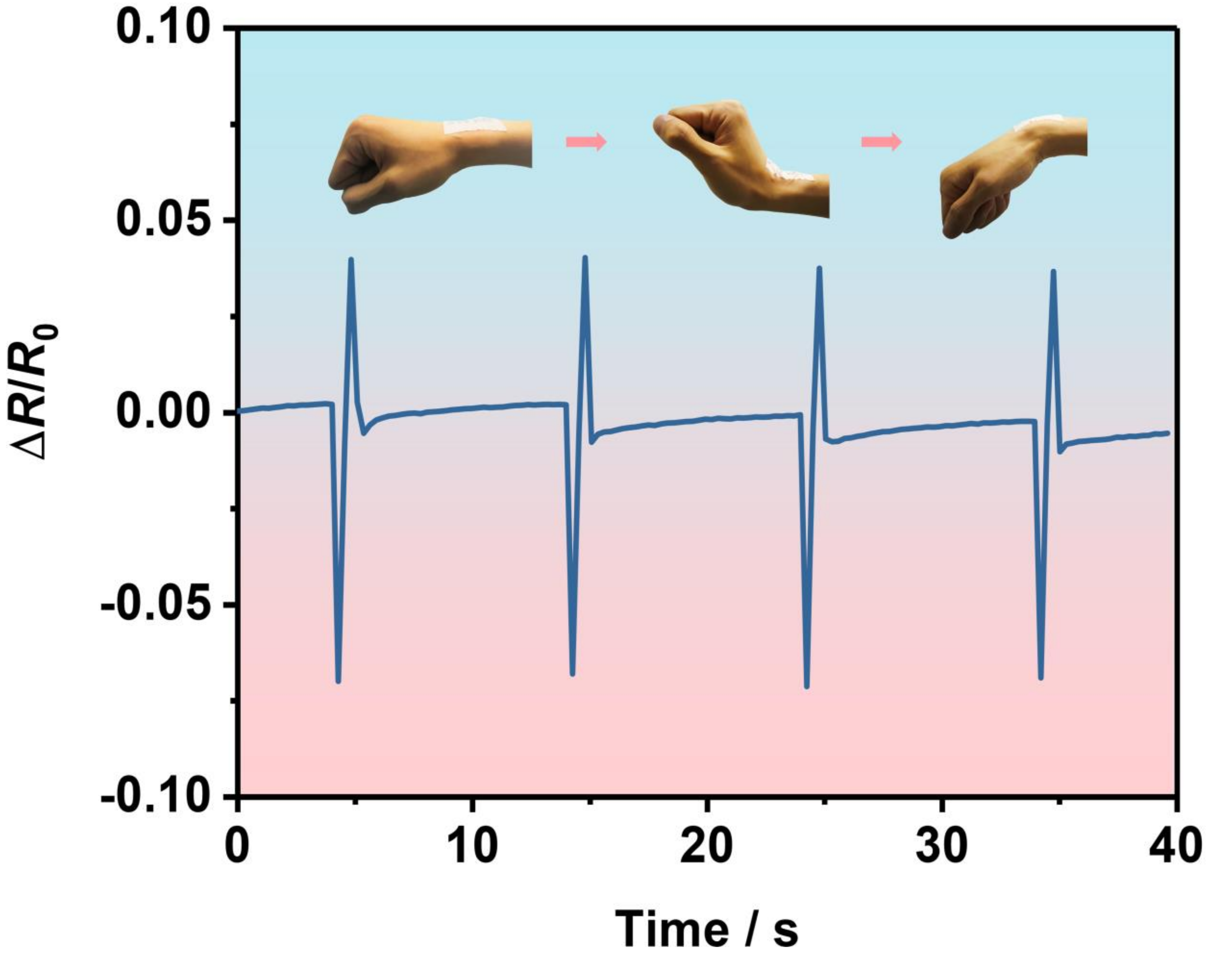

Supplementary Fig. 17 Demonstration of human motion detection with printed hydrogel sensor.

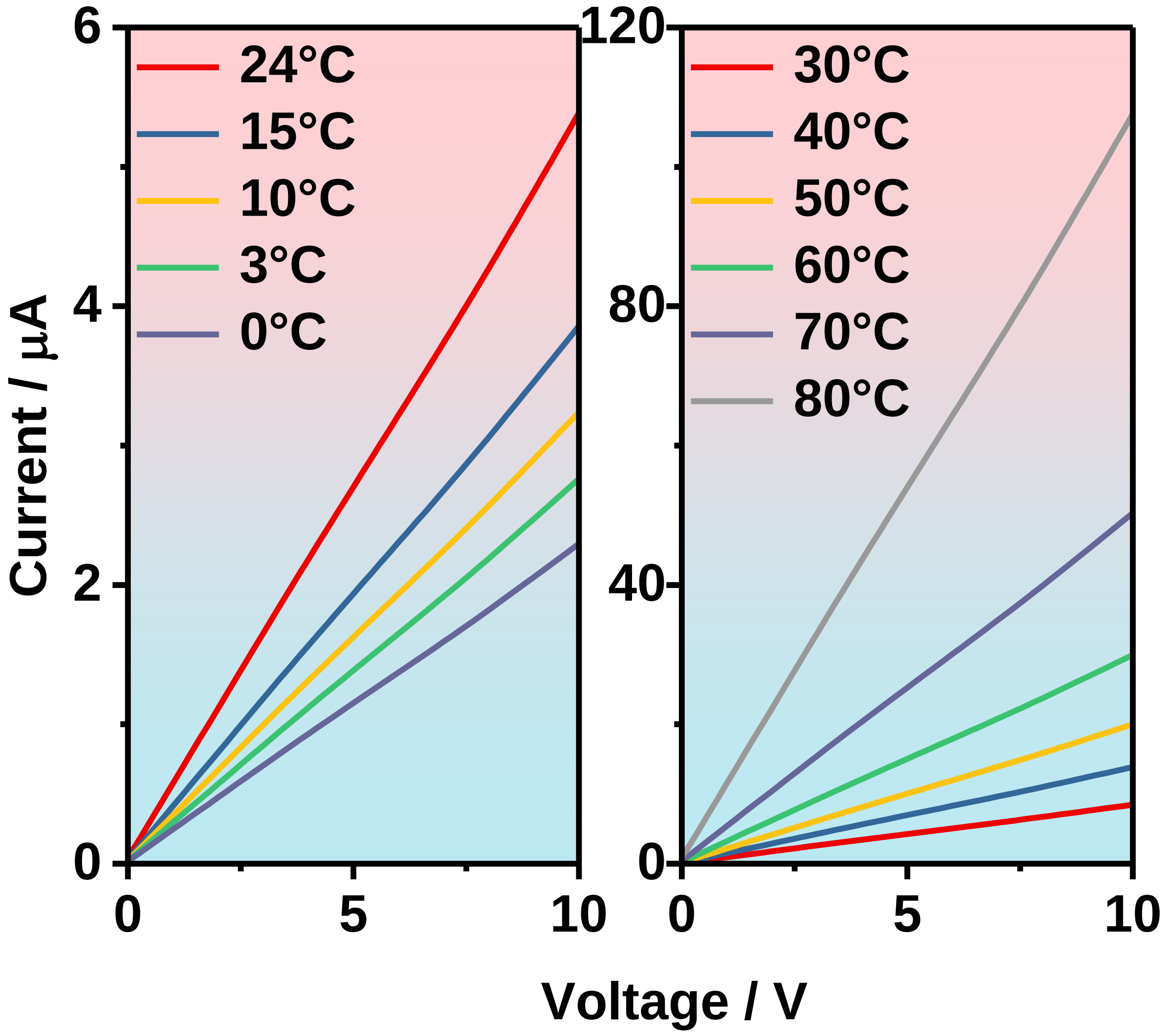

Supplementary Fig. 18 Variation of hydrogel conductivities under different temperature fields.

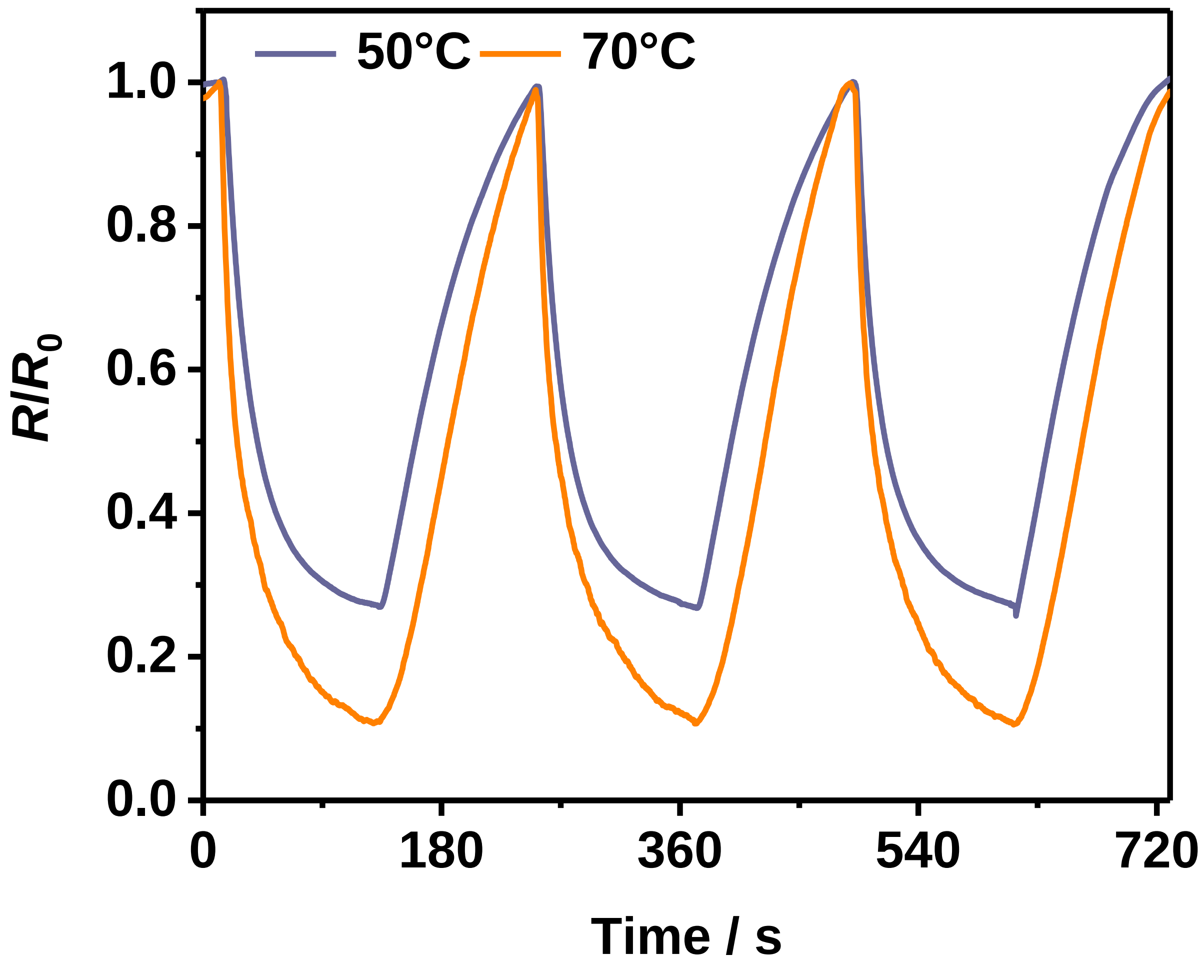

Supplementary Fig. 19 Resistance change ratio (R/R_0) under 50 °C and 70 °C (starting temperature: T_s, *i.e.*, 24 °C).

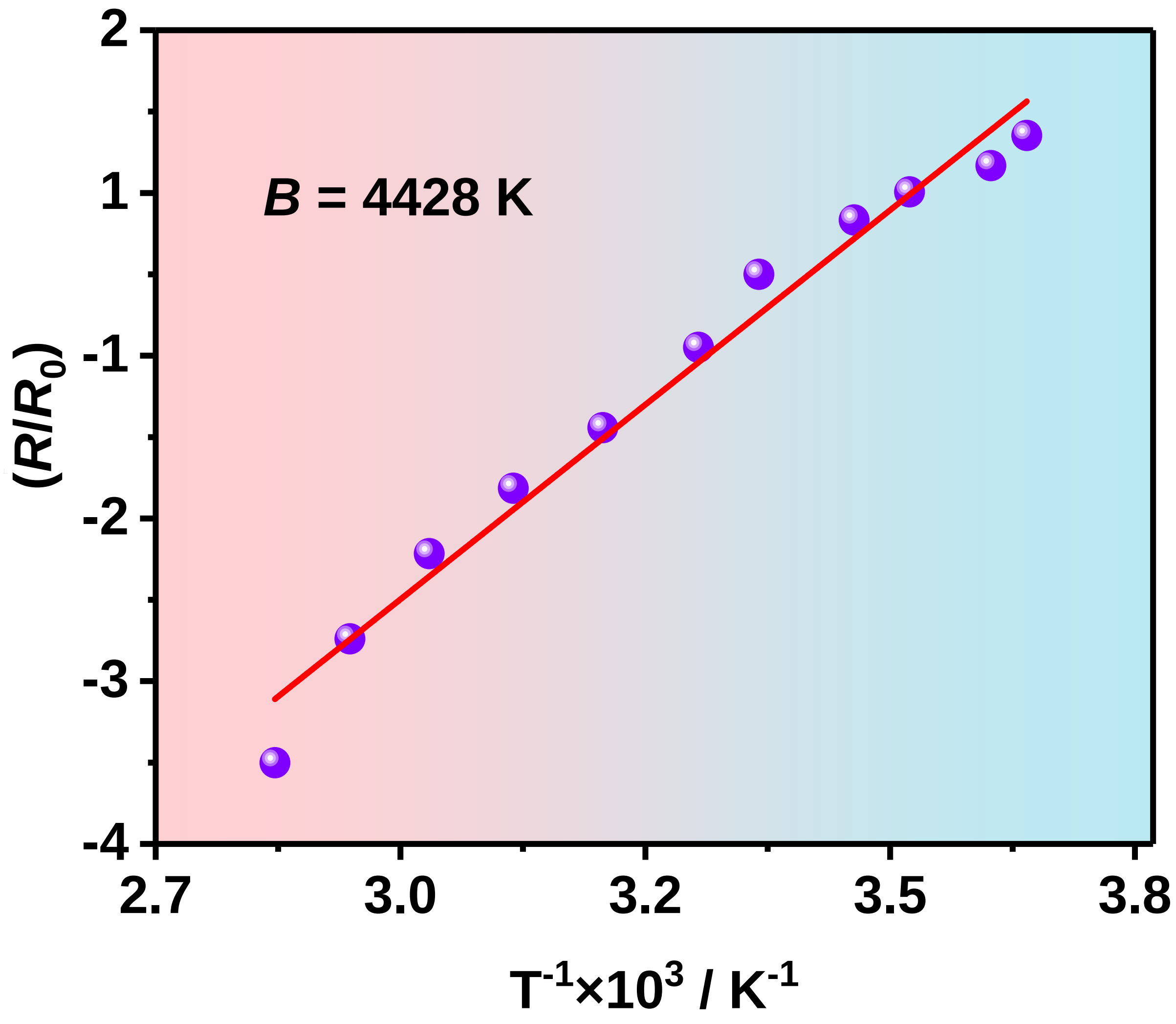

Supplementary Fig. 20 *B* value of printed thermistor ranging from 0 °C to 80 °C (starting temperature: T_s, *i.e.*, 24 °C).

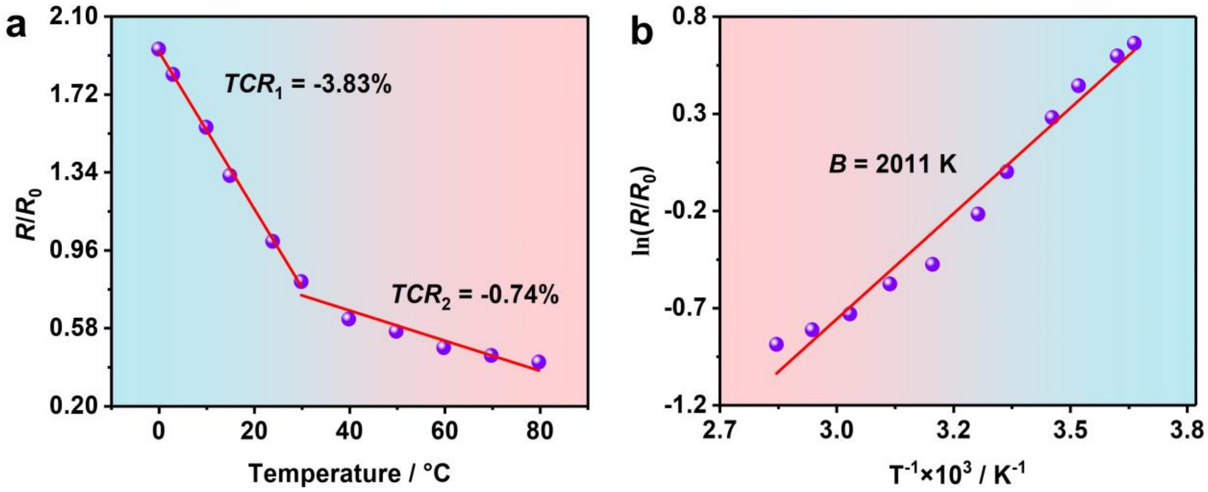

Supplementary Fig. 21 a Temperature coefficient of resistance (*TCR*) and **b** *B* values of MXenes unloaded hydrogel ranging from 0 °C to 80 °C (starting temperature: T_s, *i.e.*, 24 °C).

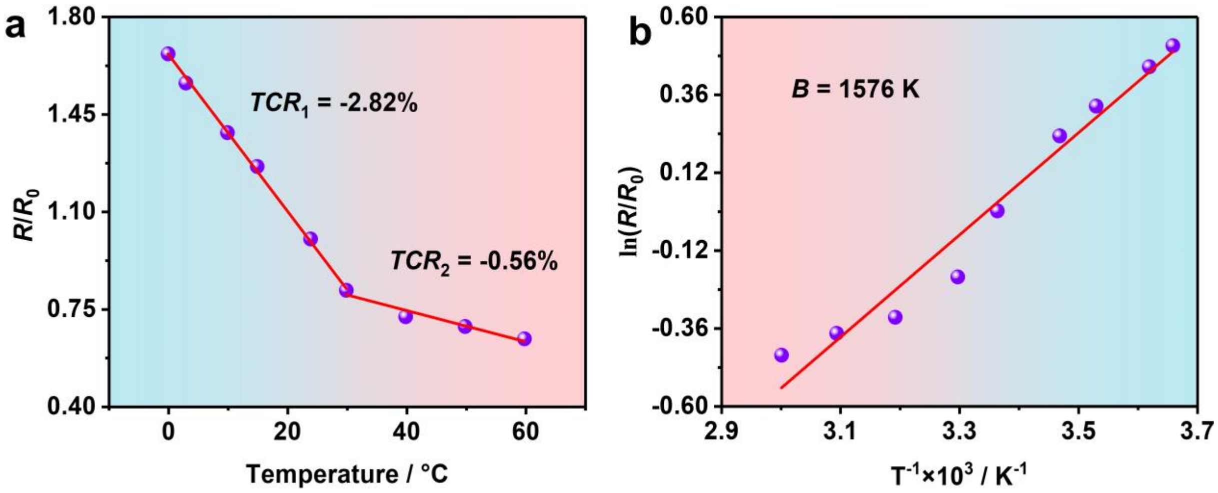

Supplementary Fig. 22 a Temperature coefficient of resistance (*TCR*) and **b** *B* values of MXenes and glycerol unloaded hydrogel ranging from 0 °C to 60 °C (starting temperature: T_s, *i.e.*, 24 °C).

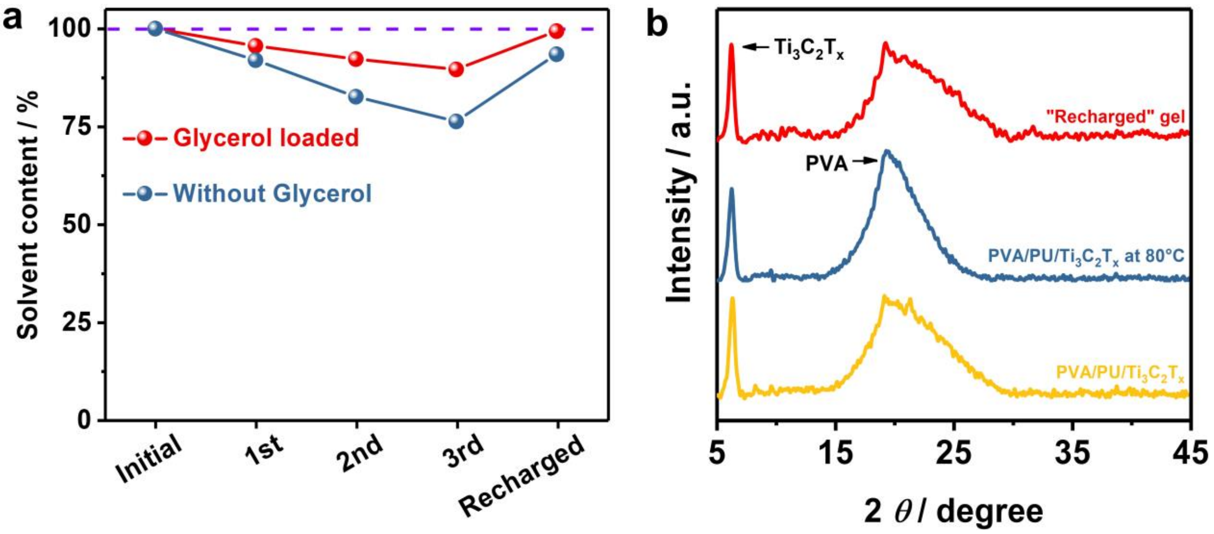

Supplementary Fig. 23 **a** The effect of glycerol on solvent content (water retention) of hydrogel during 3 heating-cooling cycles and “recharging” process. **b** X-ray diffraction (XRD) patterns of hydrogel under different circumstance.

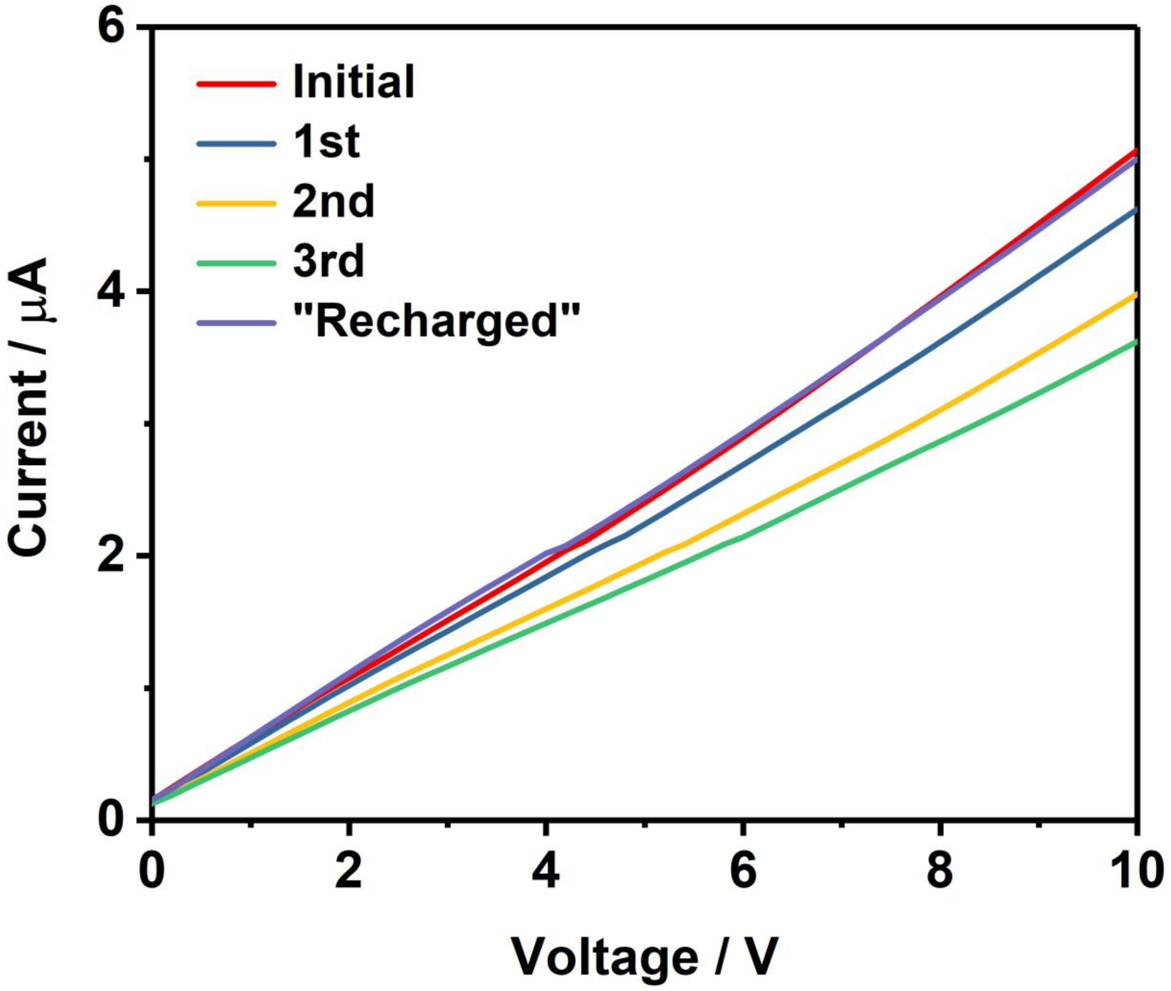

Supplementary Fig. 24 The conductivity of printed thermistor during 3 heating-cooling cycles and "recharging" process.

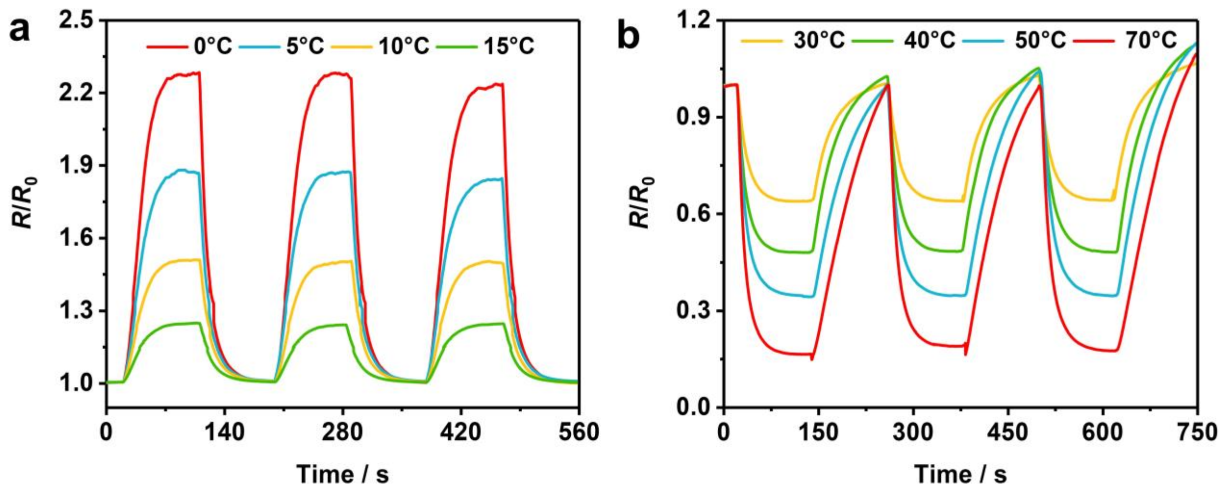

Supplementary Fig. 25 Resistance change ratio (R/R_0) of "recharged" hydrogel under various temperatures (starting temperature: T_s, *i.e.*, 24 °C). **a** R/R_0 change below the starting temperature, **b** R/R_0 change above the starting temperature.

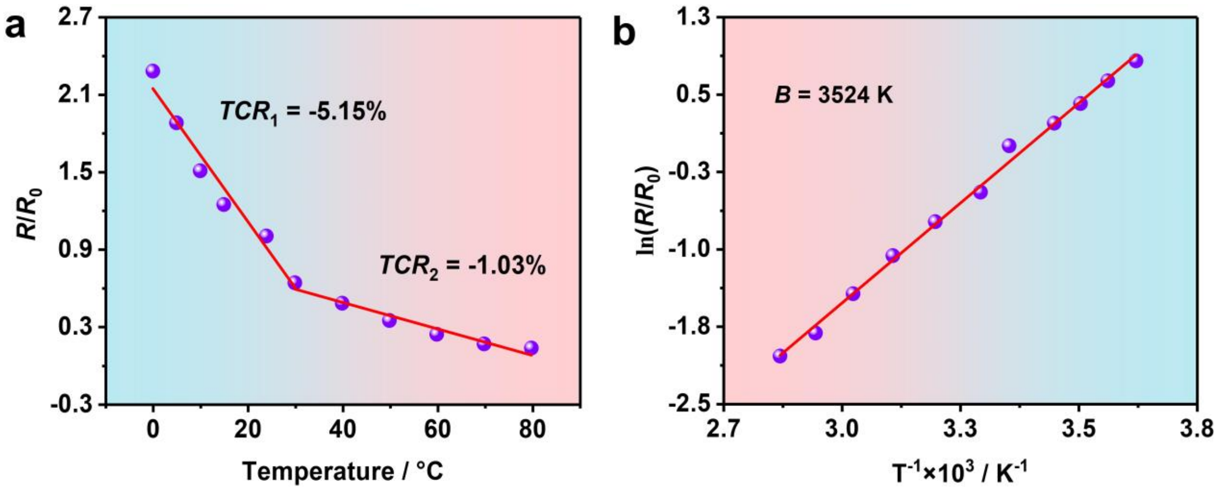

Supplementary Fig. 26 a Temperature coefficient of resistance (*TCR*) and **b** *B* values of “recharged” thermistor.

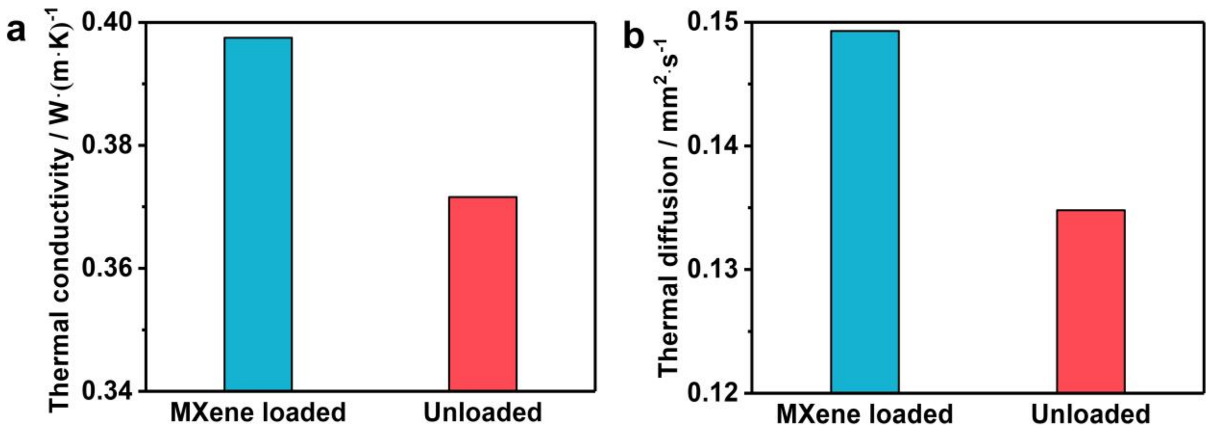

Supplementary Fig. 27 The effects of $Ti_3C_2T_x$ on **a** thermal conductivity and **b** thermal diffusion of hydrogel.

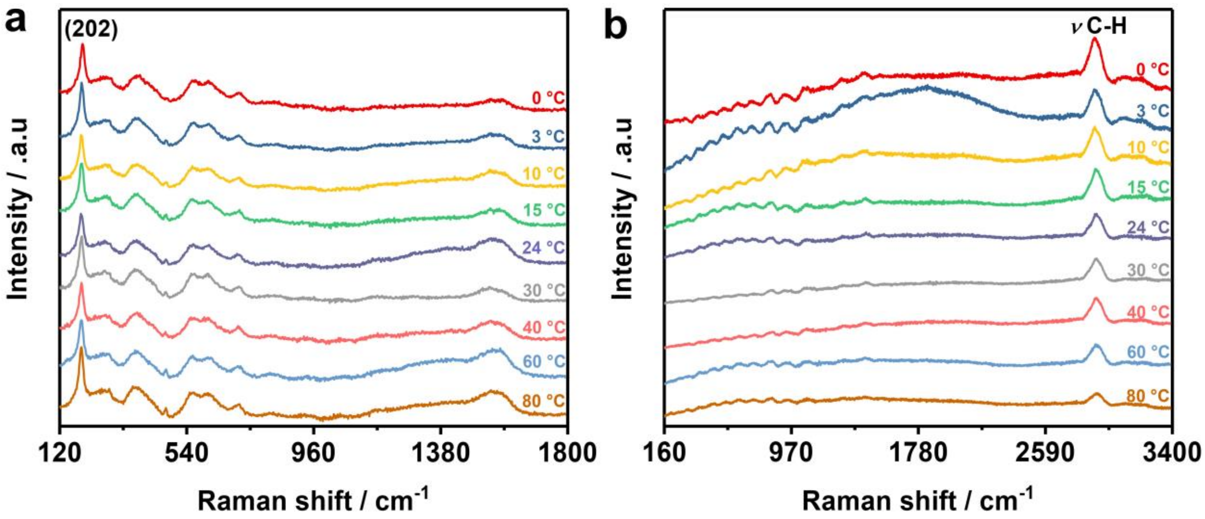

Supplementary Fig. 28 Temperature-dependent Raman spectra of **a** $Ti_3C_2T_x$ and **b** $Ti_3C_2T_x$ loaded hydrogel at variable temperature from 0 °C to 80 °C.

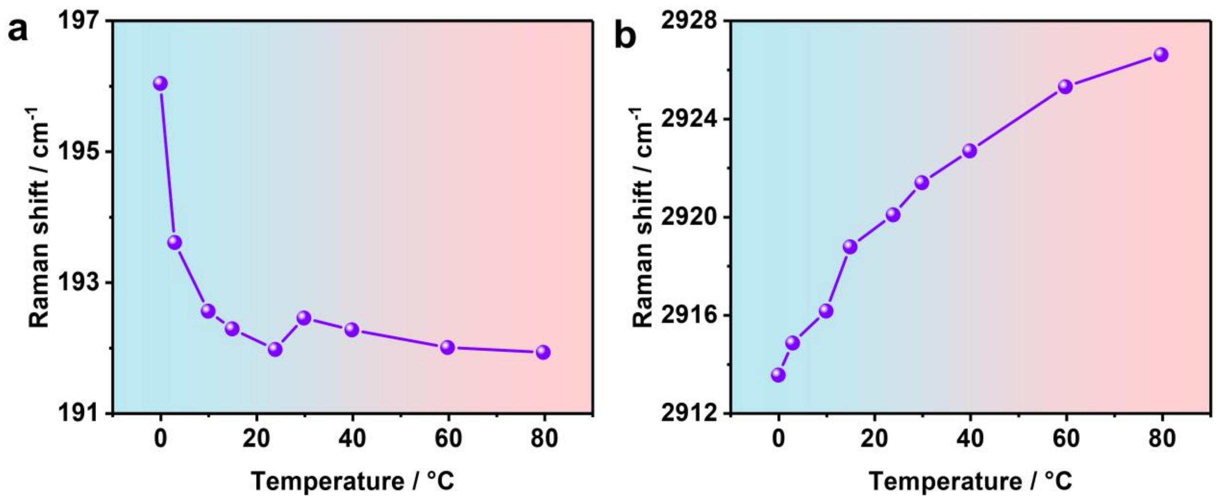

Supplementary Fig. 29 Raman shifts of **a** the (202) peak of $Ti_3C_2T_x$ spectra and **b** $Ti_3C_2T_x$ loaded hydrogel at variable temperature from 0 °C to 80 °C.

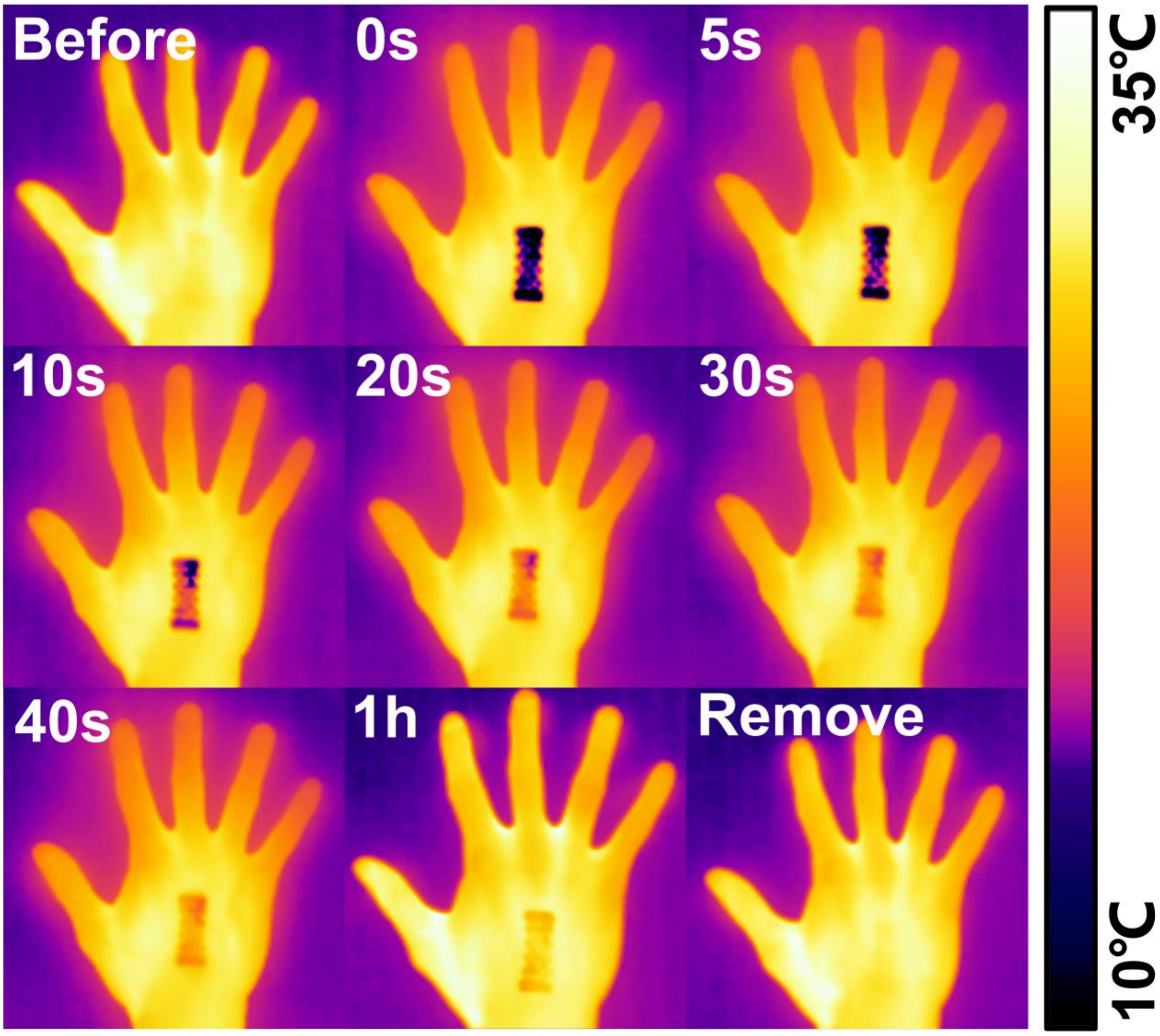

Supplementary Fig. 30 Thermal images of the thermistor attached on the skin.

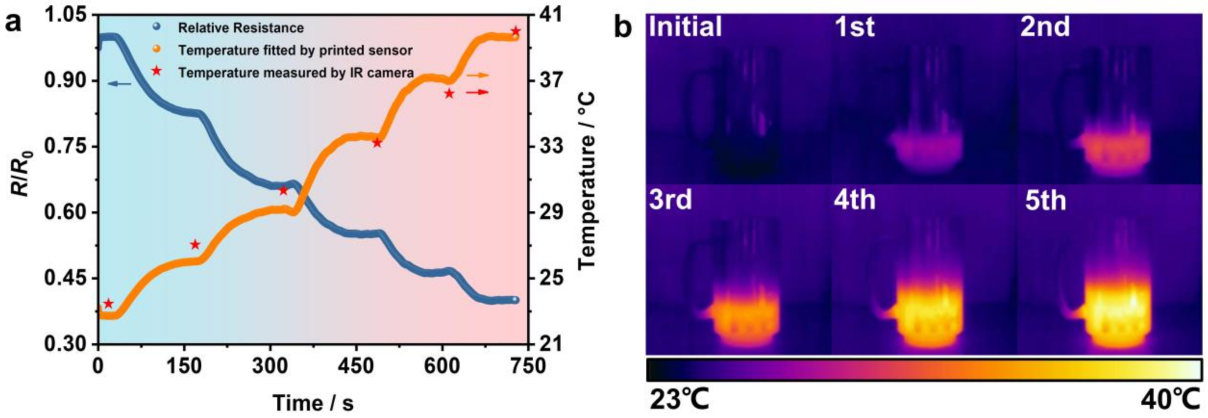

Supplementary Fig. 31 a Resistance change ratio (R/R_0), fitted temperature results and temperature recorded by infra (IR) camera. **b** IR thermal images of thermistor attached cup contained water.

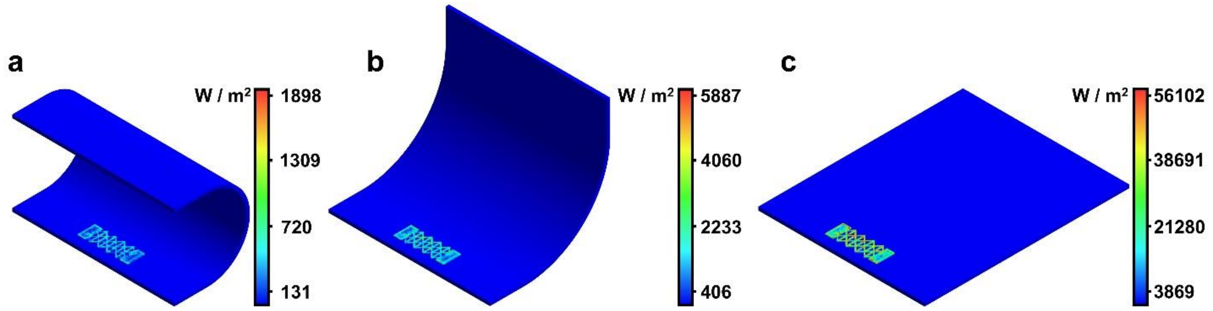

Supplementary Fig. 32 Finite element analysis of heat flow at **a** initial state, **b** semi-deployed state and **c** end state of deployment.

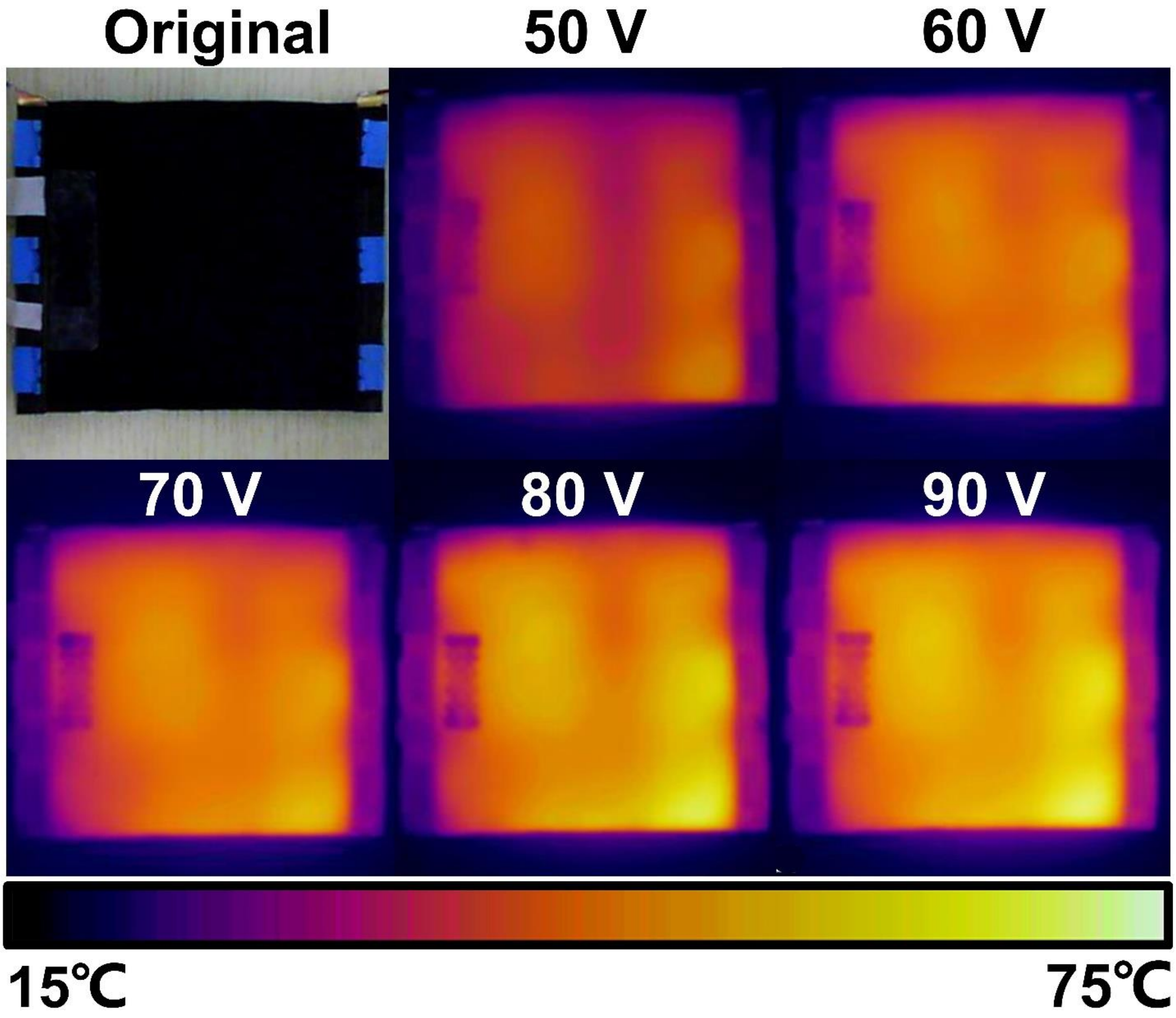

Supplementary Fig. 33 The actual surface temperature distributions at end state under variable voltages.

Supplementary Table 1 Performance comparison of thermistors.

Sensing materials	TCR (% / °C)	Working range (°C)	Ref.
PEDOT : PSS	−0.03	30−45	[11]
AgNPs / polyimine	0.17	24−54	[12]
BP / LEG / SEBS	0.1736	25−50	[13]
Pt NFs / SF / PU	0.205	20−60	[14]
CNTs / SNF	0.52	26−110	[15]
graphene-doped PU fibers	−0.815	20−100	[16]
P(DMAPS-co-AA) / Al^{3+}	−1.6	25−50	[17]
XSBR/SSCNT	−1.636	30−100	[18]
PANI NFs / PAA	−1.64	40−110	[19]
S-RGOH	−2.04	26−101	[20]
P(AAm-AAc)	−2.89	15−25	[21]
	−1.27	25−40	
MXenes / PU / PVA	−5.27	0−30	This work
	−1.11	30−80	

Abbreviations: PEDOT : PSS, Poly(3,4-ethylenedioxythiophene)-poly(styrene sulfonate); AgNPs, Ag nanoparticles; BP, black Phosphorus; LEG, laser-engraved graphene; SEBS, polystyrene-block-poly(ethylene-ran-butylene)-block polystyrene; Pt NFs, Pt nanofibers; SF, silk fibroins; CNTs, carbon nanotubes; SNF, silk nanofiber; S-RGOH, sulfonated reduced graphene oxide hydrogel; P(DMAPS-co-AA), poly(3-dimethyl(methacryloyloxyethyl) ammonium propane sulfonate-co-acrylic acid);

XSBR, carboxylic styrene butadiene rubber; SSCNT, hydrophilic sericin non-covalently modified carbon nanotubes; P(AAm-AAc), poly(acrylamide-acrylic acid).

Supplementary References

1. Feng, Y. *et al.* Muscle-inspired MXene conductive hydrogels with anisotropy and low-temperature tolerance for wearable flexible sensors and arrays. *Adv. Funct. Mater.* **31**, 2105264, (2021).
2. Ge, G. *et al.* $Ti_3C_2T_x$ MXene-activated fast gelation of stretchable and self-healing hydrogels: a molecular approach. *ACS Nano* **15**, 2698-2706, (2021).
3. He, S. *et al.* Non-swelling and anti-fouling MXene nanocomposite hydrogels for underwater strain sensing. *Adv. Mater. Technol.* 2101343, (2021).
4. Li, S. *et al.* Environmentally stable, mechanically flexible, self-adhesive, and electrically conductive $Ti_3C_2T_x$ MXene hydrogels for wide-temperature strain sensing. *Nano Energy* **90**, 106502, (2021).
5. Li, X. *et al.* Healable, degradable, and conductive MXene nanocomposite hydrogel for multifunctional epidermal sensors. *ACS Nano* **15**, 7765-7773, (2021).
6. Liu, J. *et al.* Additive manufacturing of Ti_3C_2-MXene-functionalized conductive polymer hydrogels for electromagnetic-interference shielding. *Adv. Mater.* **34**, 2106253, (2022).
7. Wei, H. *et al.* Ultrastretchable, highly transparent, self-adhesive, and 3D-printable ionic hydrogels for multimode tactical sensing. *Chem. Mater.* **33**, 6731-6742, (2021).
8. Wei, Y. *et al.* MXene-based conductive organohydrogels with long-term environmental stability and multifunctionality. *Adv. Funct. Mater.* **30**, 2005135, (2020).

9. Xu, K. *et al.* Ultradurable noncovalent cross-linked hydrogels with low hysteresis and robust elasticity for flexible electronics. *Chem. Mater.* **34**, 7, (2022).

10. Zhang, X. *et al.* Adhesive ionohydrogels based on ionic liquid/water binary solvents with freezing tolerance for flexible ionotronic devices. *Chem. Mater.* **34**, 1065-1077, (2022).

11. An, B. W., Heo, S., Ji, S., Bien, F. & Park, J. U. Transparent and flexible fingerprint sensor array with multiplexed detection of tactile pressure and skin temperature. *Nat. Commun.* **9**, 2458, (2018).

12. Zou, Z. *et al.* Rehealable, fully recyclable, and malleable electronic skin enabled by dynamic covalent thermoset nanocomposite. *Sci. Adv.* **4**, eaaq0508, (2018).

13. Chhetry, A. *et al.* Black phosphorus@laser-engraved graphene heterostructure-based temperature–strain hybridized sensor for electronic-skin applications. *Adv. Funct. Mater.* **31**, 2007661, (2021).

14. Huang, J. *et al.* Stretchable and heat-resistant protein-based electronic skin for human thermoregulation. *Adv. Funct. Mater.* **30**, 1910547, (2020).

15. Gogurla, N., Kim, Y., Cho, S., Kim, J. & Kim, S. Multifunctional and ultrathin electronic tattoo for on-skin diagnostic and therapeutic applications. *Adv. Mater.* **33**, 2008308, (2021).

16. Hu, X. *et al.* Multiscale disordered porous fibers for self-sensing and self-cooling integrated smart sportswear. *ACS Nano* **14**, 559-567, (2020).

17. Tan, Y. *et al.* Dual cross-linked ion-based temperature-responsive conductive hydrogels with multiple sensors and steady electrocardiogram monitoring. *Chem. Mater.* **32**, 7670-7678, (2020).

18. Lin, M. *et al.* A high-performance, sensitive, wearable multifunctional sensor based on

rubber/CNT for human motion and skin temperature detection. *Adv. Mater.* **34**, 2107309, (2021).

19. Ge, G. *et al.* Muscle-inspired self-healing hydrogels for strain and temperature sensor. *ACS Nano* **14**, 218-228, (2020).

20. Wu, J. *et al.* Self-calibrated, sensitive, and flexible temperature sensor based on 3D chemically modified graphene hydrogel. *Adv. Electron. Mater.* **7**, 2001084, (2021).

21. Park, T. H. *et al.* Highly sensitive on-skin temperature sensors based on biocompatible hydrogels with thermoresponsive transparency and resistivity. *Adv. Healthcare Mater.* **10**, 2100469, (2021).
