# Supplementary material for: Approaching intrinsic dynamics of MXenes hybrid hydrogel for 3D printed multimodal intelligent devices with ultrahigh superelasticity and temperature sensitivity
Source: Nat Commun. 2022 Jun 14;13:3420. doi: 10.1038/s41467-022-31051-7 (PMC9197829; doi:10.1038/s41467-022-31051-7)
Supplement: Supplementary file 3 — Description of Additional Supplementary Files [file 41467_2022_31051_MOESM3_ESM.pdf]

Description of Additional Supplementary Files

File Name: Supplementary Movie 1
Description: Video of DIW printing process.

File Name: Supplementary Movie 2
Description: Infrared thermal video of deployment processes for ESMP system.
