# Supplementary material for: Approaching intrinsic dynamics of MXenes hybrid hydrogel for 3D printed multimodal intelligent devices with ultrahigh superelasticity and temperature sensitivity
Source: Nat Commun. 2022 Jun 14;13:3420. doi: 10.1038/s41467-022-31051-7 (PMC9197829; doi:10.1038/s41467-022-31051-7)
Supplement: Supplementary file 2 — Peer Review File [file 41467_2022_31051_MOESM2_ESM.pdf]

nature portfolio

Peer Review File

Approaching Intrinsic Dynamics of MXenes Hybrid Hydrogel for 3D Printed Multimodal Intelligent Devices with Ultrahigh Superelasticity and Temperature sensitivity

Open Access This file is licensed under a Creative Commons Attribution 4.0 International License, which permits use, sharing, adaptation, distribution and reproduction in any medium or format, as long as you give appropriate credit to the original author(s) and the source, provide a link to the Creative Commons license, and indicate if changes were made. In the cases where the authors are anonymous, such as is the case for the reports of anonymous peer reviewers, author attribution should be to 'Anonymous Referee' followed by a clear attribution to the source work. The images or other third party material in this file are included in the article's Creative Commons license, unless indicated otherwise in a credit line to the material. If material is not included in the article's Creative Commons license and your intended use is not permitted by statutory regulation or exceeds the permitted use, you will need to obtain permission directly from the copyright holder. To view a copy of this license, visit http://creativecommons.org/licenses/by/4.0/.

Reviewers' Comments:

Reviewer #1:
Remarks to the Author:
The authors did a very interesting work. They prepared MXenes hybrid hydrogel based on polyurethane/polyvinyl alcohol by 3D printing technology. In addition, the mechanical properties of the hydrogel can be further controlled by adding glycerin. It be used as a very stable and sensitive temperature sensor. The author also carefully studied the influence of MXene and glycerol content on the properties of the hydrogel. And through the combination of simulation and theory, the internal dynamics of MXenes and glycerin in the hydrogel stretching process were explored. There are some key issues needed to be addressed before further consideration.
1. In the title, it is mentioned that this is a superelastic hydrogel, however, there is only elastic modulus in the article, and lack of other characteristics of elasticity, such as rheological test, compression recovery test, etc. And there is no comparison with other superelastic hydrogels reported in the literature.
2. In line 122, "which is in accordance with the thickness of $Ti_3C_2T_x$ bilayer structure", related literature with MXene size characterization should be cited.
3. In Supplementary Fig. 5b, with the increased of glycerol, the elastic modulus increased first, then decreased. Why?
4. MXene is the only conductive medium in the hydrogel. When the temperature changes, the relative resistance of the hydrogel also changes. Please explain in detail how temperature affects the conductivity of MXene.
5. Due to it is a hydrogel for temperature sensing, the hydrogel will inevitably lose water at high temperatures (even with the addition of the humectant glycerin). What is the water content of the hydrogel after being placed at a higher temperature for a period of time? Is there any significant change in the performance of the hydrogel?
6. The hydrogel is mainly composed of freeze-thaw PVA. In the temperature sensing test, a relatively stable signal can be maintained even at 80°C, but as far as we know, the microcrystalline area of the PVA has begun to be destroyed at this temperature. It will inevitably affect the performance of the sensor. Why does the hydrogel maintain its stability?
7. Why use polyurethane to improve the mechanical properties of PVA hydrogels? The specific role of polyurethane in the hydrogel system?
8. The application experiment on temperature sensing is too single, and some temperature sensing applications that are combined with reality need to be added.

Reviewer #2:
Remarks to the Author:
In this article, the authors have prepared a hydrogel in which MXenes are bonded to polyurethane and polyvinyl alcohol (PU/PVA) and they measured its strain and temperature sensing performance.

Although the manuscript has some interesting results and nice figures, this reviewer thinks it presents only evolutionary rather than revolutionary progress in the field of MXene hydrogels. MXene hydrogels have been reported for strain and temperature sensing and using PVA as well as glycerol. Also, unlike the authors' claim, mechanistic explanations for MXene hydrogel behavior have been discussed openly in the literature, particularly in relation to their strain sensing and self-healing behavior.

As such, I do not believe the manuscript meets the threshold of novelty and groundbreaking discoveries required by nature communications. It deserves to be published, but not in this journal.

Here are more specific comments which the authors can use to revise the manuscript before they submit it elsewhere
• The MXene hydrogel performance is not as great as the authors claim. For example, hydrogels with

much better GF and strain limit have been reported. The authors need to compare their results to existing ones focusing on MXene-containing hydrogels

•The authors claim that previous studies on MXene hydrogels are ambiguous and do not adequately explain the MXene-hydrogel formation mechanism. It seems the same can be said of the authors themselves.

o For example, the mechanistic interaction of each one of the additives (PVA, PU, glycerol) in the hydrogel (chemical bonding, chain entanglement) are highly speculative and the authors provide no spectroscopic or physical analysis to prove their claims about the role of each component in the MXene hydrogel formation process
o The authors claim "the relatively flexible PVA chains with abundant polar hydroxyl groups promotes the internal friction induced strain hysteresis". Can the authors back up these statements with experimental evidence
o The authors write "However, the adding of glycerol promotes the hydrogen bonding between the solvents and solutes, which not only locks the water in the system, but also enhances the chains interaction between PU and PVA, ensuring the water retention during heating process and the stability of PVA crystalline domains". Can you please provide some evidence

• It is known that the pH and ion concentration can change strain-resistance behavior of MXene hydrogel. Do these factors come into play after adding glycerol which the authors claim produces more anions (presumably OH)
• What is the water content of the hydrogel? How does that change with temperature for samples with an without glycerol. This has to be measured
• What is the conductivity of MXene itself?
• The authors claim significant hydrogen bond formation. This normally has strong impact on self-healing performance of eth hydrogel. The authors should discuss the self-healing performance of the hydrogel and how it compares to other MXene hydrogels
• The authors are missing many important publications on MXene hydrogel used for sensing

Reviewer #3:
Remarks to the Author:
The authors present a new method for generating 3D Printable multimodal devices based on hybrid systems of 2D Mxenes and hydrogels. They investigated the intrinsic dynamics, printability and first applications in sensor devices with ultrahigh super-elasticity and temperature sensitivity as a technical basis for flexible sensors.
While the basic constituent MXenes and hydrogels of the hydrid systems discussed here are worldwide investigated, the authors combined specific physical properties of these starting systems in a clever way, such that a novel emergent quality was generated in the composite system. In addition, the direct 3D ink writing capability of their MXene bonded hydrogels allowed them the prepare complex functional devices structures, thus overcoming conventional machining. In particular, a strain sensor system with excellent temperature sensitivity based on this concept, displaying excellent cycling stability as required for practical application was demonstrated.
In essence, the authors demonstrate a novel concept for combining the properties of conductive nanoparticular MXene flakes with those of hydrogels systems by tailoring both, the electrical properties and mechanical flexibility making the whole system compatible for a variety of new applications in flexible and printable electronics. A major problem to overcome was the ambiguity in the response of MXene bonded PU/PVA hydrogel-based hydrogel sensors, which was resolved by optimizing the loadings of the MXenes by glycerol. Mechanical properties and sensitivity of the devices were simulated by finite element analysis. Temperature dependent Raman spectroscopy was performed to evaluate the temperature response of thermally induced tunneling effects in the hydrogel sensor. In this way, the transport mechanism of the designed strain sensing system could be

evaluated in detail.
The manuscript provides clear and detailed description of the fabrication process and to components used.
The authors also gave a detailed report on the morphological structure of the hydrogel with and without the MXene loading. Elemental analysis is displayed to demonstrate the uniformity of the inorganic constituents od the hybrid material, while structural analysis done by FESEM and AFM revealed the morphology of the overall systems (FESEM/AFM). Further valuable structural data were optioned by XRD and photoelectron spectroscopy (XPS), the latter allowing to evaluate the bonding situation of the compound system, for example, oxidation occurring after delamination. Finally, Gauge factors, strain sensing performance and thermal stability were investigated and optimized. Electron tunneling through the breakpoints of the conducting network was identified as the major transport mechanism in the new strain sensors. Since electron tunneling is extremely sensitive to distance variation, a very high gauge factor of the devices could be observed. In fact, several thousand stretching-release cycles could be demonstrated at 10% /5Hz strain, which is significantly higher than in conventional metallic systems. As a result, the authors pointed out that their new strain sensor has a high potential to be applied for reliably detecting human motion, for example, in human health monitoring.
Finally, a refinement of the tunneling effect is proposed in the ms by making use of thermally-assisted tunneling and the fact that Hydrogels used are good thermal conductors allowing to transfer thermal energy from the skin to the sensor. As a result, the system acts an efficient thermistor. As proposed by the authors, this, in combination with an appropriate electro-responsive shape memory polymer system may form a basis for constructing a hinge system for adjusting solar cells.
Detailed descriptions of the technical concepts used for the experiments and computer simulations are given in the methods-chapter complemented by original data including two animations in the SI. The comprehensive set of data presented by the authors consortium fully support their claims in the manuscript. The references in the manuscript reflect the state of previous work well.
The concept presented by the authors is highly valuable both with respect to the basic science involved and in view of the careful engineering behind it. The manuscript is well written and the data supporting the results and conclusions are of excellent quality.
To summarize, I recommend the publication of this manuscript in Nature Communications after a minor correction in Fig. 1:
To make the terminology in Fig. 1 clearer for readers outside the field, i.e. which specific type of MXene was used here, the labelling ‘Ti3C2Tx/LiCl’ (center of the top row of Fig 1) should be better replaced by Ti3C2Fx/LiCl, since LiF/HCL etching was used here, see J. Zhao et al., Mater. Chem. A, 2018, 6, 24031.

Response to referees letter

Reviewer #1:

The authors did a very interesting work. They prepared MXenes hybrid hydrogel based on polyurethane/polyvinyl alcohol by 3D printing technology. In addition, the mechanical properties of the hydrogel can be further controlled by adding glycerin. It be used as a very stable and sensitive temperature sensor. The author also carefully studied the influence of MXene and glycerol content on the properties of the hydrogel. And through the combination of simulation and theory, the internal dynamics of MXenes and glycerin in the hydrogel stretching process were explored. There are some key issues needed to be addressed before further consideration.

Response to Reviewer #1

We deeply appreciate the positive comments of the reviewer, which encouraged us for further optimization.

1. In the title, it is mentioned that this is a superelastic hydrogel, however, there is only elastic modulus in the article, and lack of other characteristics of elasticity, such as rheological test, compression recovery test, etc. And there is no comparison with other superelastic hydrogels reported in the literature.

Response

Thanks for the comment. Affected by the Covid-19 epidemic, the rheological tests have to be suspended, but other characteristics including dynamic mechanical analysis (DMA) and compression recovery tests of the hydrogel have been conducted. DMA results shows that both storage modulus and

loss modulus can be obviously increased through loading $Ti_3C_2T_x$ flakes, which endows the hydrogel with superior anti-deformation ability and elasticity, detailed discussion was described in the revised manuscript and supplementary information (**Manuscript, Page 11, Line 186 to 200. Supplementary Information, Page 12**). In addition, the introducing of PU, glycerol and $Ti_3C_2T_x$ all enhanced the compressive strength, presenting similar the deformation-recovery behaviors. Detailed discussion was described in the revised manuscript and supplementary information (**Manuscript, Page 10, Line 169 to 170, Line 173 to 177, Line 178 to 181, Page 11, Line 182 to 186. Supplementary Information, Page 8 to 10**). The comparison with other superelastic hydrogels with MXene reported in the literature shows that the prepared gel in this work performs superb mechanical strength, which has been summarized in the revised manuscript and supplementary information (**Manuscript, Page 10, Line 178 to 181. Supplementary Information, Page 11, Supplementary Fig. 10b**).

2. In line 122, "which is in accordance with the thickness of $Ti_3C_2T_x$ bilayer structure", related literature with MXene size characterization should be cited.

Response

Thanks for the valuable suggestion. The size characterization results of MXene sheets have been analyzed in several published works, and related literatures of Ref. 33, Ref. 35 to 38 report that the thickness of monolayer $Ti_3C_2T_x$ flakes is less than 1.5 nm, which have been cited in the revised manuscript to support the relevant conclusion of this work[1-5] (**Manuscript, Page 8, Line 117 to 122**).

3. In Supplementary Fig. 5b, with the increased of glycerol, the elastic modulus increased first, then decreased. Why?

Response

Thanks for the question. The gelation should have been maintained at -20 °C, but the previous actual gelation temperature of some samples for stretching tests could be slightly higher than -20 °C due to the refrigeration equipment maintenance, resulting in insufficient crystallization of PVA chains for samples with 50 wt% glycerol and 0.5 wt% $Ti_3C_2T_x$, respectively. In order to correct the experimental results for avoiding misleading, the authors re-measured the tensile strength and elastic modulus of the hydrogel samples, the results present that the tensile strength and strain range can be gradually enhanced with the increased of glycerol loading, relevant discussion was described in the revised manuscript and supplementary information (**Manuscript, Page 10, Line 173 to 177. Supplementary Information, Page 9, Supplementary Fig. 8a and f**). Besides, the stretching performance of 0.5 wt% $Ti_3C_2T_x$ hydrogel was weakened compared with 0.25 wt% and 0.1 wt% $Ti_3C_2T_x$ loading, but a continuously rising trend can be noticed from the result of the elastic modulus, detailed discussion was presented in the revised manuscript and supplementary information (**Manuscript, Page 10, Line 178 to 181, Page 11, Line 182 to 186. Supplementary Information, Page 10, Supplementary Fig. 9a, Page 11, Supplementary Fig. 10a**).

4. MXene is the only conductive medium in the hydrogel. When the temperature changes, the relative resistance of the hydrogel also changes. Please explain in detail how temperature affects the conductivity of MXene.

Response

Thanks for the comment. The prepared $Ti_3C_2T_x$ flakes in this work were assembled to a homogeneous membrane with 7 μm thickness through vacuum filtration, and the conductivity was measured at 7.257

$\times$ 10^4 $S \cdot m^{-1}$, presenting extraordinary electrical conductivity, which was described in the revised manuscript (**Manuscript, Page 8, Line 119 to 122**). Furthermore, the effect of temperature on the conductivity of MXene mainly depends on the electronic properties and microscopic configuration of $Ti_3C_2T_x$. According to the band structure analysis, bare Ti_3C_2 exhibits metallicity, while surface functionalized $Ti_3C_2T_x$ presents narrow bandgap semiconducting properties[6,7]. The presence of T groups (-F and -OH) results in the transformation of metallic Ti_3C_2 into $Ti_3C_2T_x$ with bandgaps of 0.05 eV and 0.1 eV, respectively. In addition, the T groups bonded with Ti atoms are located in hollow sites (type I) above adjacent carbon atoms, exhibiting semiconductive properties[8,9]. If oriented on the topmost of the C atoms, the materials are metallic (type II). Generally, Type I is relatively more energetically stable for $Ti_3C_2T_x$. Based on the narrow bandgap semiconductive behavior, heat endows electrons with sufficient energy to achieve transitions and boost carrier concentration. Therefore, the resultant conductivity of MXene can be heightened with the increasing temperature, the detailed discussion has been analyzed in the revised manuscript (**Manuscript, Page 22, Line 411 to 423**).

5. Due to it is a hydrogel for temperature sensing, the hydrogel will inevitably lose water at high temperatures (even with the addition of the humectant glycerin). What is the water content of the hydrogel after being placed at a higher temperature for a period of time? Is there any significant change in the performance of the hydrogel?

Response

Thanks for the questions. At first, the solvent content at 54% relative humidity under room temperature within 20 days of hydrogel with and without glycerol was measured, revealing that the water retention ability can be promoted through loading glycerol, which has been discussed in the revised manuscript and supplementary information (**Manuscript, Page 9, Line 159, Page 10, Line 160 to 163.**

Supplementary Information, Page 7). Besides, the dehydration of hydrogel is inevitable under high temperatures, the solvent content and the conductivity of the gel after each heating-cooling cycle was measured, the results showed that minor dehydration occurred and the conductivity decreased slightly during 3 heating-cooling cycles at high temperatures, detailed discussion was described in the revised manuscript and supplementary information (**Manuscript, Page 19, Line 350 to 354, Page 20, Line 372 to 375**). However, after re-immersed in glycerol aqueous solution for 120 s, the solvent content and the conductivity of dehydrated hydrogel with glycerol loaded was “recharged” to the original state, and the temperature sensing behaviors of “recharged” gel were measured, showing an extremely close level compared with the original performance from Fig. 4d. Therefore, the printed hydrogel is capable of “recharging” like commercial rechargeable battery for reusing in temperature sensing, relevant discussion was provided in the revised manuscript and supplementary information (**Manuscript, Page 19, Line 354 to 358, Page 20, Line 372 to 383, Page 21, Line 384 to 385. Supplementary Information, Page 23, Supplementary Fig. 23a, Page 25 to 27**).

6. The hydrogel is mainly composed of freeze-thaw PVA. In the temperature sensing test, a relatively stable signal can be maintained even at 80°C, but as far as we know, the microcrystalline area of the PVA has begun to be destroyed at this temperature. It will inevitably affect the performance of the sensor. Why does the hydrogel maintain its stability?

Response

Thanks for the question. The phenomenon about the dissolution of the PVA microcrystalline domains also appeared in this work. As shown in Supplementary Fig. 22, the upper limit of the temperature response range of the hydrogel without glycerin only stays at 60 °C, and the gel can be dissolved due to

the destruction of the microcrystalline domains over 60 °C. The XRD patterns of glycerol loaded gel revealed that the PVA crystal domains can be preserved after loading PU and $Ti_3C_2T_x$ flakes before heating-cooling cycles, which was analyzed in the revised manuscript and supplementary information (**Manuscript, Page 9, Line 149 to 153. Supplementary Information, Page 5**). Then, the XRD spectrum of glycerol and $Ti_3C_2T_x$ loaded gel under various circumstances were presented in Supplementary Fig. 23b, the results presented that the crystallization peak of PVA was preserved after 3 heating-cooling cycles and the following "recharging", meaning that the presence of glycerol avoids the gel dissolution, indicating that the microcrystalline domains in PVA can be protected, and the detailed analysis has been discussed in the revised manuscript and supplementary information (**Manuscript, Page 19, Line 358 to 359, Page 20, Line 360 to 371. Supplementary Information, Page 24, Supplementary Fig. 23b**).

7. Why use polyurethane to improve the mechanical properties of PVA hydrogels? The specific role of polyurethane in the hydrogel system?

Response

Thanks for the valuable questions. Commercial waterborne polyurethane (PU) is possessed with easy processing and eco-friendliness, and the increasingly wide application requirements promote the continuous maturity of scaled production technology. PU not only presents relatively remarkable mechanical strength and chemical stability, but also exhibits good compatibility with PVA in aqueous solution, therefore it is suitable for modifications about water-soluble polymers. PU chains are composed of soft domains and hard domains, showing a unique microphase separation structure. The soft domains determine the elasticity and low temperature resistance, and the hard domains act as

physical crosslink sites, dominating the mechanical strength of the materials. Specifically, the interaction mechanism of each component including PU of the hydrogel system was investigated through XRD and Fourier transform infrared spectroscopy (FTIR) analysis. The XRD patterns of glycerol loaded gel prove that the crystal structure of PVA can be preserved after loading PU and $Ti_3C_2T_x$, which was discussed in the revised manuscript and supplementary information in detail (**Manuscript, Page 9, Line 149 to 153. Supplementary Information, Page 5**). Meanwhile, FTIR of the pristine PVA/H_2O hydrogel successively loaded with glycerol, PU and $Ti_3C_2T_x$ presents the obvious wavenumber shift of −OH groups and C−O bond, proving the formation of hydrogen bonds. Therefore, based on the PVA crystal domains framework, PU chains not only form physical entanglement with the PVA chains, but also provide polar groups to generate hydrogen bonds with other components, endowing the hydrogel with superb mechanical properties, which was analyzed in the revised manuscript and supplementary information (**Manuscript, Page 9, Line 153 to 158. Supplementary Information, Page 6**).

8. The application experiment on temperature sensing is too single, and some temperature sensing applications that are combined with reality need to be added.

Response

Thanks for the suggestion. Owing to the superior and stable thermal sensing performance, the printed thermistor can be used for detecting surface temperature of objects in real time. The sensor was attached to the bottom of cup outside wall with 40 mL cool water (24 °C) contained, then 15 mL hot water (90 °C) was successively added for 5 times. The increased temperature can be recorded through R/R_0 change, the gradual decline of R/R_0 can be observed after adding hot water each time, and the

obtained temperature from R/R_0 change was calculated according to TCR results from Fig. 4d, which is close to the temperature illustration of commercial IR camera, meaning that the printed thermistor can truly present the actual temperature change of the measured object, and the detailed discussion has been supplied in the revised manuscript and supplementary information (**Manuscript, Page 22, Line 426 to 433, Supplementary Information, Page 32**).

Reviewer #2:

In this article, the authors have prepared a hydrogel in which MXenes are bonded to polyurethane and polyvinyl alcohol (PU/PVA) and they measured its strain and temperature sensing performance. Although the manuscript has some interesting results and nice figures, this reviewer thinks it presents only evolutionary rather than revolutionary progress in the field of MXene hydrogels. MXene hydrogels have been reported for strain and temperature sensing and using PVA as well as glycerol. Also, unlike the authors' claim, mechanistic explanations for MXene hydrogel behavior have been discussed openly in the literature, particularly in relation to their strain sensing and self-healing behavior. As such, I do not believe the manuscript meets the threshold of novelty and groundbreaking discoveries required by nature communications. It deserves to be published, but not in this journal. Here are more specific comments which the authors can use to revise the manuscript before they submit it elsewhere.

Response to Reviewer #2

We are grateful to the reviewer for the comments, which encouraged us for further optimization. As common eco-friendly materials, PVA and glycerol have indeed been used in the field of MXene hydrogel sensing. However, the significance of this work lies in the fabrication of physically crosslinked superelastic hydrogel with toughness and temperature resistance, meanwhile, cost-effective processing of such classical materials through structure-specialized 3D printing technology was further developed, revealing the mechanical behaviors by means of finite element analysis. Furthermore, the temperature sensing performance was emphasized in detail with clarifying the response mechanism through in-situ characterization techniques. Meanwhile, the application scenarios about the aerospace field were developed for the first time.

1. The MXene hydrogel performance is not as great as the authors claim. For example, hydrogels with much better GF and strain limit have been reported. The authors need to compare their results to existing ones focusing on MXene-containing hydrogels.

Response

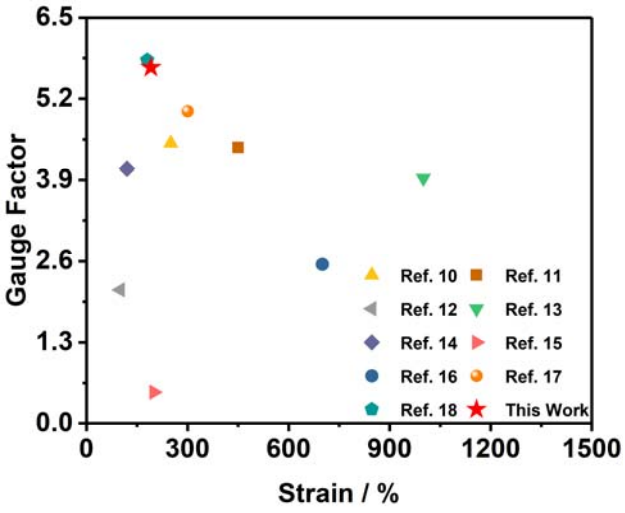

Fig. R1 Comparison of GF with reported literatures.

Thanks for the suggestion. Several studies have reported higher GF and wider strain range for MXene hydrogels, the comparison is presented in Fig. R1[10-18]. As the authors describe in the manuscript, the printed hydrogel exhibits stable strain-responsive behavior. What this work highlight is that achieving cost-effective structure-specialization of common materials through additive manufacturing technology, GF and strain limit can be controlled according to the printing pattern for meeting the requirements of various strain application scenarios, detailed discussion was demonstrated in the revised manuscript and supplementary information (**Manuscript, Page 13, Line 234 to 237. Supplementary Information, Page 15)**. Besides, compared with the bulk hydrogel fabricated by the traditional casting method, the printed multi triangle patterned hydrogel device can be possessed with superior breathability and heat dissipation when attached on human skin, which has been discussed in the revised manuscript and supplementary information (**Manuscript, Page 22, Line 424 to 426. Supplementary Information, Page 31**).

2. The authors claim that previous studies on MXene hydrogels are ambiguous and do not adequately explain the MXene-hydrogel formation mechanism. It seems the same can be said of the authors themselves.

Response

Thanks for the comment. Previous reports have elaborated the formation mechanism of MXene hydrogels, including freeze crystallization, ionic complexation, dynamic covalent cross-linking and free radical polymerization. However, the relevant stimuli-responsive mechanism still needs to be further explored, which encourage the authors to finish in-depth investigation in this work.

2.1 For example, the mechanistic interaction of each one of the additives (PVA, PU, glycerol) in the hydrogel (chemical bonding, chain entanglement) are highly speculative and the authors provide

no spectroscopic or physical analysis to prove their claims about the role of each component in the MXene hydrogel formation process.

Response

Thanks for the comment. The XRD patterns of glycerol loaded gel with various components were identified first, showing that the crystal structure can be preserved after loading PU and $Ti_3C_2T_x$, detailed discussion was analyzed in the revised manuscript and supplementary information (**Manuscript, Page 9, Line 149 to 153, Supplementary Information, Page 5**). Besides, the Fourier transform infrared spectroscopy (FTIR) of the pristine PVA/H_2O hydrogel successively loaded with glycerol, PU and $Ti_3C_2T_x$ was performed. The obvious wavenumber shift of −OH groups and C−O bond can be observed, which proves the formation of hydrogen bonds. Hence the gelation can be contributed by PVA crystal domains and hydrogen bonding among each component, the detailed discussion was analyzed in the revised manuscript and supplementary information (**Manuscript, Page 9, Line 153 to 158, Supplementary Information, Page 6**). In addition, it has been reported that the absorption energy of PVA with water and glycerol molecules was calculated through density functional theory (DFT)[12]. The results showed that the combination of PVA and binary solvent presented a lower energy state than the one-component solvent system, indicating that the PVA chains can be more stable in the coexistence of water and glycerol and form additional hydrogen bonds. Generally, polymer chains are oriented randomly in a homogeneous composites system, as a consequence, chain entanglements tend to be formed during cross-linking[19].

2.2 The authors claim "the relatively flexible PVA chains with abundant polar hydroxyl groups promotes the internal friction induced strain hysteresis". Can the authors back up these statements with experimental evidence?

Response

Thanks for the question. The dynamic mechanical analysis (DMA) and compression recovery tests of the hydrogel have been conducted. DMA results shows that both storage modulus and loss modulus can be obviously increased through loading $Ti_3C_2T_x$ flakes, which endows the hydrogel with superior anti-deformation ability and elasticity, detailed discussion was described in the revised manuscript and supplementary information (**Manuscript, Page 11, Line 186 to 200. Supplementary Information, Page 12**). In addition, the introducing of PU, glycerol and $Ti_3C_2T_x$ all enhanced the compressive strength, presenting similar the deformation-recovery behaviors. Detailed discussion was described in the revised manuscript and supplementary information (**Manuscript, Page 10, Line 169 to 170, Line 173 to 177, Line 178 to 181, Page 11, Line 182 to 186. Supplementary Information, Page 8 to 10**). Specifically, the results of pristine PVA/H_2O hydrogel showed that obvious hysteresis circles can be noticed (**Supplementary Information, Page 8, Supplementary Fig. 7a**). According to the theory of polymer high elasticity and viscoelasticity, as described below. The hysteresis of polymers is related to the chemical structure of chains, and the hysteresis of rigid chains is significantly weaker than that of flexible chains. The occurrence of hysteresis is often accompanied by the appearance of mechanical loss. The external force needs to change the chain configurations and overcome the intrinsic friction during the movement of chains when polymer stretching or compressing. Retracting the force, the deformed chains re-coil with frictional resistance being offset during chains motion. Generally, with great frictional resistance comes to serious hysteresis and mechanical loss, the latter also depends on the chains structure. Polymer chains with fewer side groups present less frictional resistance and mechanical loss, but the side group with stronger polarity will lead to the opposite result. Therefore, the PVA chains are relatively flexible, but a large number of polar groups (hydroxyl) lead to serious

hysteresis and mechanical loss, which has been supplied into the manuscript (**Manuscript, Page 16, Line 283 to 293**). For brevity, the statement of "the relatively flexible PVA chains with abundant polar hydroxyl groups promotes the internal friction induced strain hysteresis" has been replaced by "The hysteresis resulted from PVA chains still occurs during deformation-recovery cycles" (**Manuscript, Page 16, Line 297**).

2.3 The authors write "However, the adding of glycerol promotes the hydrogen bonding between the solvents and solutes, which not only locks the water in the system, but also enhances the chains interaction between PU and PVA, ensuring the water retention during heating process and the stability of PVA crystalline domains". Can you please provide some evidence?

Response

Thanks for the question. FTIR spectrum presented the obvious wavenumber shift of −OH groups and C−O bond, proving that hydrogen bonding formation among each component of the hydrogel, detailed discussion was described in the revised manuscript and supplementary information (**Manuscript, Page 9, Line 153 to 158. Supplementary Information, Page 6**). Besides, XRD patterns of glycerol loaded hydrogel at the end of the 3 heating-cooling cycles presented that the PVA crystal domains can be preserved (**Manuscript, Page 19, Line 358 to 359, Page 20, Line 360 to 371. Supplementary Information, Page 24, Supplementary Fig. 23b**). Meanwhile, the effect of glycerol on solvent content within 20 days of hydrogel was investigated, the high water retention of glycerol loaded gel ensures the stability of the hydrogel thermistor, which was discussed in the revised manuscript and supplementary information (**Manuscript, Page 9, Line 159, Page 10, Line 160 to 163. Supplementary Information, Page 7**). Although minor dehydration occurred during heating-cooling cycles, the gel can be "recharged" through immersing in glycerol aqueous solution for 120 s, with the

recovery of solvent content and conductivity, detailed discussion was described in the revised manuscript and supplementary information (**Manuscript, Page 19, Line 350 to 358, Page 19, Line 372 to 375. Supplementary Information, Page 24, Supplementary Fig. 23a, Page 25**). Literature reported that the stability of glycerol coexisting with H_2O molecules is stronger than H_2O molecules alone, and the absorption energy of polymer with glycerol-water system is lower than which with water system, proving that hydrogen bonds can be formed[12]. In addition, Supplementary Fig. 22 demonstrates the thermal sensing performance of the hydrogel without glycerol, the working range is limited at 60 °C, as a result of the dissolution of the PVA microcrystalline domains. However, the results from Fig. 4d and Supplementary Fig. 16 exhibit that the sensing limit can be leveled to 80 °C with the presence of glycerol, meaning that the microcrystalline domains in PVA can be prevented from dissolution.

3. It is known that the pH and ion concentration can change strain-resistance behavior of MXene hydrogel. Do these factors come into play after adding glycerol which the authors claim produces more anions (presumably OH^-)?

Response

Thanks for the question. The strain sensitivity of MXene hydrogels can be affected by pH and ion concentration[10,17,20-23]. For the PVA/PU/$Ti_3C_2T_x$ hydrogel prepared in this work, the glycerol only provides sufficient hydroxyl groups as physical cross-linking sites, hence the strain-resistance behavior cannot be changed owing to the non-electrolyte performance of glycerol, which was described in the revised manuscript (**Manuscript, Page 13, Line 230 to 231**).

4. What is the water content of the hydrogel? How does that change with temperature for samples with and without glycerol? This has to be measured.

Response

Thanks for the suggestion. The solvent content at 54% relative humidity within 20 days of hydrogel with and without glycerol was measured. It is obvious that high water retention can be maintained after loading glycerol, which was analyzed in the revised manuscript and supplementary information (**Manuscript, Page 9, Line 159, Page 10, Line 160 to 163. Supplementary Information, Page 7**). In addition, the solvent content during each heating-cooling cycle results showed that minor dehydration happened to glycerol loaded gel, but severe water loss appeared for the gel without glycerol (**Manuscript, Page 19, Line 350 to 354. Supplementary Information, Page 24, Supplementary Fig. 23a**). Besides, the corresponding conductivity decreased during 3 heating-cooling cycles (**Manuscript, Page 20, Line 372 to 375. Supplementary Information, Page 25**). However, the hydrogel with glycerol can be "recharged" through immersing in glycerol aqueous solution for 120 s, the "recharged" solvent content and conductivity can be recovered to initial state. Furthermore, the temperature sensing behaviors of "recharged" gel were investigated, presenting a close level to the original performance from Fig. 4d, which means that the printed hydrogel thermistor can be reused for temperature sensing through "recharging", the detailed discussion has been demonstrated in the revised manuscript and supplementary information (**Manuscript, Page 19, Line 361 to 362, Page 20, Line 363 to 371, Supplementary Information, Page 25 to 26**).

5. What is the conductivity of MXene itself?

Response

Thanks for the question. The prepared $Ti_3C_2T_x$ flakes in this work was assembled to a homogeneous membrane with 7 μm thickness through vacuum filtration, and the conductivity was measured at 7.257 $\times 10^4$ $S \cdot m^{-1}$, presenting extraordinary electrical conductivity, which was described in the revised

manuscript (**Manuscript, Page 8, Line 119 to 122**). Furthermore, the effect of temperature on the conductivity of MXene mainly depends on the electronic properties and microscopic configuration of $Ti_3C_2T_x$. According to the band structure analysis, bare Ti_3C_2 exhibits metallicity, while surface functionalized $Ti_3C_2T_x$ presents narrow bandgap semiconducting properties[6,7]. The presence of T groups (-F and -OH) results in the transformation of metallic Ti_3C_2 into $Ti_3C_2T_x$ with bandgaps of 0.05 eV and 0.1 eV, respectively. In addition, the T groups bonded with Ti atoms are located in hollow sites (type I) above adjacent carbon atoms, exhibiting semiconductive properties[8,9]. If oriented on the topmost of the C atoms, the materials are metallic (type II). Generally, Type I is relatively more energetically stable for $Ti_3C_2T_x$. Based on the narrow bandgap semiconductive behavior, heat endows electrons with sufficient energy to achieve transitions and boost carrier concentration. Therefore, the resultant conductivity of MXene can be heightened with the increasing temperature, the detailed discussion has been analyzed in the revised manuscript (**Manuscript, Page 22, Line 411 to 423**).

6. The authors claim significant hydrogen bond formation. This normally has strong impact on self-healing performance of the hydrogel. The authors should discuss the self-healing performance of the hydrogel and how it compares to other MXene hydrogels.

Response

Thanks for the valuable suggestion. The authors had investigated the self-healing properties of hydrogels, but the self-healing effect is unobvious. The gel was cut into small pieces and put together under room temperature or heated ambient, but the gel cannot be formed completely, which could be resulted from plenty of relatively weak hydrogen bonding force provided by small glycerol molecules[14,24-26]. On the basis of the current work, the authors will carry out follow-up targeted

research.

7. The authors are missing many important publications on MXene hydrogel used for sensing.

Response

Thanks for the comment. There are various reports cited in the revised manuscript as Ref. 10, Ref. 11 and Ref. 23 to 27, investigating the MXene hydrogels for physical or chemical sensing (**Manuscript, Page 3, Line 43, Line 57**), among which the mechanical and temperature responses of the hydrogel interested the authors, but the deep understanding of response mechanism and intrinsic dynamics of MXenes Hybrid Hydrogel still need to be further explored[18,23,27-31]. The focus of this work lies in cost-effective structure-specialized processing of traditional materials, revealing the intrinsic dynamics of the system by means of theoretical simulation and in situ characterization, as well as the expansion of special applications, providing specific perspectives for the investigation of MXene hydrogels. As promising materials, the boom of MXene hydrogels will continue to support the deep research of flexible electronics, and the authors will keep concentrating on the cutting-edge progress in this field.

Reviewer #3:

The authors present a new method for generating 3D Printable multimodal devices based on hybrid systems of 2D MXenes and hydrogels. They investigated the intrinsic dynamics, printability and first applications in sensor devices with ultrahigh super-elasticity and temperature sensitivity as a technical basis for flexible sensors.

While the basic constituent MXenes and hydrogels of the hybrid systems discussed here are worldwide

investigated, the authors combined specific physical properties of these starting systems in a clever way, such that a novel emergent quality was generated in the composite system. In addition, the direct 3D ink writing capability of their MXene bonded hydrogels allowed them the prepare complex functional devices structures, thus overcoming conventional machining. In particular, a strain sensor system with excellent temperature sensitivity based on this concept, displaying excellent cycling stability as required for practical application was demonstrated.

In essence, the authors demonstrate a novel concept for combining the properties of conductive nanoparticular MXene flakes with those of hydrogels systems by tailoring both, the electrical properties and mechanical flexibility making the whole system compatible for a variety of new applications in flexible and printable electronics. A major problem to overcome was the ambiguity in the response of MXene bonded PU/PVA hydrogel-based hydrogel sensors, which was resolved by optimizing the loadings of the MXenes by glycerol. Mechanical properties and sensitivity of the devices were simulated by finite element analysis. Temperature dependent Raman spectroscopy was performed to evaluate the temperature response of thermally induced tunneling effects in the hydrogel sensor. In this way, the transport mechanism of the designed strain sensing system could be evaluated in detail.

The manuscript provides clear and detailed description of the fabrication process and to components used. The authors also gave a detailed report on the morphological structure of the hydrogel with and without the MXene loading. Elemental analysis is displayed to demonstrate the uniformity of the inorganic constituents od the hybrid material, while structural analysis done by FESEM and AFM revealed the morphology of the overall systems (FESEM/AFM). Further valuable structural data were optioned by XRD and photoelectron spectroscopy (XPS), the latter allowing to evaluate the bonding

situation of the compound system, for example, oxidation occurring after delamination. Finally, Gauge factors, strain sensing performance and thermal stability were investigated and optimized. Electron tunneling through the breakpoints of the conducting network was identified as the major transport mechanism in the new strain sensors. Since electron tunneling is extremely sensitive to distance variation, a very high gauge factor of the devices could be observed. In fact, several thousand stretching-release cycles could be demonstrated at 10%/5Hz strain, which is significantly higher than in conventional metallic systems. As a result, the authors pointed out that their new strain sensor has a high potential to be applied for reliably detecting human motion, for example, in human health monitoring.

Finally, a refinement of the tunneling effect is proposed in the ms by making use of thermally-assisted tunneling and the fact that Hydrogels used are good thermal conductors allowing to transfer thermal energy from the skin to the sensor. As a result, the system acts an efficient thermistor. As proposed by the authors, this, in combination with an appropriate electro-responsive shape memory polymer system may form a basis for constructing a hinge system for adjusting solar cells.

Detailed descriptions of the technical concepts used for the experiments and computer simulations are given in the methods-chapter complemented by original data including two animations in the SI. The comprehensive set of data presented by the authors consortium fully support their claims in the manuscript. The references in the manuscript reflect the state of previous work well.

The concept presented by the authors is highly valuable both with respect to the basic science involved and in view of the careful engineering behind it. The manuscript is well written and the data supporting the results and conclusions are of excellent quality.

To summarize, I recommend the publication of this manuscript in Nature Communications after a

minor correction in Fig. 1:

To make the terminology in Fig. 1 clearer for readers outside the field, i.e., which specific type of MXene was used here, the labelling "$Ti_3C_2T_x$/LiCl" (center of the top row of Fig 1) should be better replaced by $Ti_3C_2F_x$/LiCl, since LiF/HCL etching was used here, see Zhao et al., J. Mater. Chem. A, 2018, 6, 24031.

Response to Reviewer #3

We deeply appreciate the careful review and positive comments of the reviewer, inspired by the suggestion and the literature provided, the labelling "$Ti_3C_2T_x$/LiCl" has been replaced by "$Ti_3C_2F_x$/LiCl" (**Manuscript, Page 5, Line 78**).

References

1. Alhabeb, M. *et al.* Guidelines for synthesis and processing of two-dimensional titanium carbide ($Ti_3C_2T_x$ MXene). *Chem. Mater.* **29**, 7633-7644, (2017).
2. Ding, L. *et al.* MXene molecular sieving membranes for highly efficient gas separation. *Nat. Commun.* **9**, 155, (2018).
3. Lu, Z., Wu, Y., Ding, L., Wei, Y. & Wang, H. A lamellar MXene ($Ti_3C_2T_x$)/PSS composite membrane for fast and selective lithium-ion separation. *Angew. Chem., Int. Ed.* **60**, 22265-22269, (2021).
4. Wang, J. *et al.* Highly conductive MXene film actuator based on moisture gradients. *Angew. Chem., Int. Ed.* **59**, 14029-14033, (2020).
5. Yun, T. *et al.* Electromagnetic shielding of monolayer MXene assemblies. *Adv. Mater.* **32**, 1906769, (2020).

6. Naguib, M. *et al.* Two-dimensional nanocrystals produced by exfoliation of Ti_3AlC_2. *Adv. Mater.* **23**, 4248-4253, (2011).

7. Sinha, A. *et al.* MXene: an emerging material for sensing and biosensing. *TrAC, Trends Anal. Chem.* **105**, 424-435, (2018).

8. Zhang, X., Zhang, Z. & Zhou, Z. MXene-based materials for electrochemical energy storage. *J. Energy Chem.* **27**, 73-85, (2018).

9. Pei, Y. *et al.* $Ti_3C_2T_x$ MXene for sensing applications: recent progress, design principles, and future perspectives. *ACS Nano* **15**, 3996-4017, (2021).

10. Hao, F. *et al.* Bifunctional smart hydrogel dressing with strain sensitivity and NIR-responsive performance. *ACS Appl. Mater. Interfaces* **13**, 46938-46950, (2021).

11. He, S. *et al.* Non-swelling and anti-fouling MXene nanocomposite hydrogels for underwater strain sensing. *Adv. Mater. Technol.*, 2101343, (2021).

12. Hu, C. *et al.* Stable, strain-sensitive conductive hydrogel with antifreezing capability, remoldability, and reusability. *ACS Appl. Mater. Interfaces* **10**, 44000-44010, (2018).

13. Li, S.-N. *et al.* Environmentally stable, mechanically flexible, self-adhesive, and electrically conductive $Ti_3C_2T_x$ MXene hydrogels for wide-temperature strain sensing. *Nano Energy* **90**, 106502, (2021).

14. Wang, Z. *et al.* Antiliquid-interfering, antibacteria, and adhesive wearable strain sensor based on superhydrophobic and conductive composite hydrogel. *ACS Appl. Mater. Interfaces* **13**, 46022-46032, (2021).

15. Zhang, J. *et al.* Highly stretchable and self-healable MXene/polyvinyl alcohol hydrogel electrode for wearable capacitive electronic skin. *Adv. Electron. Mater.* **5**, 1900285, (2019).

16. Zhao, W., Zhang, D., Yang, Y., Du, C. & Zhang, B. A fast self-healing multifunctional polyvinyl alcohol nano-organic composite hydrogel as a building block for highly sensitive strain/pressure sensors. *J. Mater. Chem. A* **9**, 22082-22094, (2021).

17. Zhu, Y. *et al.* Multifunctional $Ti_3C_2T_x$ MXene composite hydrogels with strain sensitivity toward absorption-dominated electromagnetic-interference shielding. *ACS Nano* **15**, 1465-1474, (2021).

18. Feng, Y. *et al.* Muscle-inspired MXene conductive hydrogels with anisotropy and low-temperature tolerance for wearable flexible sensors and arrays. *Adv. Funct. Mater.* **31**, 2105264, (2021).

19. Kim, J., Zhang, G., Shi, M. & Suo, Z. Fracture, fatigue, and friction of polymers in which entanglements greatly outnumber cross-links. *Science* **374**, 212-216, (2021).

20. Deng, Y. *et al.* Fast gelation of $Ti_3C_2T_x$ MXene initiated by metal ions. *Adv. Mater.* **31**, 1902432, (2019).

21. Lee, K. H. *et al.* Muscle fatigue sensor based on $Ti_3C_2T_x$ MXene hydrogel. *Small Methods* **5**, 2100819, (2021).

22. Li, X. *et al.* Healable, degradable, and conductive MXene nanocomposite hydrogel for multifunctional epidermal sensors. *ACS Nano* **15**, 7765-7773, (2021).

23. Li, Y., Yan, J., Liu, Y. & Xie, X. Super tough and intelligent multibond network physical hydrogels facilitated by $Ti_3C_2T_x$ MXene nanosheets. *ACS Nano* **16**, 1567-1577, (2022).

24. Caprioli, M. *et al.* 3D-printed self-healing hydrogels via digital light processing. *Nat. Commun.* **12**, 2462, (2021).

25. Khatib, M., Zohar, O. & Haick, H. Self-healing soft sensors: from material design to

implementation. *Adv. Mater.* **33**, 2004190, (2021).

26. Wang, S. & Urban, M. W. Self-healing polymers. *Nat. Rev. Mater.* **5**, 562-583, (2020).

27. Liu, J. *et al.* Additive manufacturing of $Ti_3C_2T_x$-MXene-functionalized conductive polymer hydrogels for electromagnetic-interference shielding. *Adv. Mater.* **34**, 2106253, (2022).

28. Li, G. *et al.* Development of conductive hydrogels for fabricating flexible strain sensors. *Small* **18**, 2101518, (2022).

29. Guan, L. *et al.* Balloon inspired conductive hydrogel strain sensor for reducing radiation damage in peritumoral organs during brachytherapy. *Adv. Funct. Mater.,* 2112281, (2022).

30. Luo, X. *et al.* A flexible multifunctional triboelectric nanogenerator based on MXene/PVA hydrogel. *Adv. Funct. Mater.* **31**, 2104928, (2021).

31. Wei, Y. *et al.* MXene-based conductive organohydrogels with long-term environmental stability and multifunctionality. *Adv. Funct. Mater.* **30**, 2005135, (2020).

Reviewers' Comments:

Reviewer #1:
Remarks to the Author:
The authors have fully addressed the reviewer's comments and replied to all the questions, now I recommend the publication of this manuscript.

Reviewer #2:
Remarks to the Author:
I think the manuscript may now be published.

Reviewer #3:
Remarks to the Author:
With the improvements and additional information provided by the authors in their revised manuscript, I recommend publication in Nature Communications in its present form.

Reviewer #1:

The authors have fully addressed the reviewer's comments and replied to all the questions, now I recommend the publication of this manuscript.

Response to Reviewer #1

We deeply appreciate the reviewing efforts and positive comments of the reviewer, which encourages us for further exploration in this field.

Reviewer #2:

I think the manuscript may now be published.

Response to Reviewer #2

We are grateful to the reviewer for the reviewing and comments, which has significantly improved the presentation of our manuscript.

Reviewer #3:

With the improvements and additional information provided by the authors in their revised manuscript, I recommend publication in Nature Communications in its present form.

Response to Reviewer #3

We appreciate the reviewer's positive evaluation of our work, which encourages us for follow-up research in this field.
